# Multifunctional ion-conductive polymer coatings for high-performance sulfide solid-state batteries with Ni-rich cathodes†

**DOI:** 10.1039/d5ta01827g

**Published:** 2025-05-13

**Authors:** Pranav Karanth, Jelle H. Prins, Ajay Gautam, Zhu Cheng, Jef Canals-Riclot, Swapna Ganapathy, Pierfrancesco Ombrini, Alix Ladam, Sebastien Fantini, Marnix Wagemaker, Fokko M. Mulder

**Affiliations:** a Department of Chemical Engineering, Delft University of Technology The Netherlands F.m.mulder@tudelft.nl; b Department of Radiation Science and Technology, Delft University of Technology The Netherlands; c Solvionic 11 Chemin des Silos Toulouse 31100 France

## Abstract

Sulfide-based solid-state batteries (SSBs) are emerging as a top contender for next-generation rechargeable batteries with improved safety and higher energy densities. However, SSBs with Ni-rich cathode materials such as LiNi_0.82_Mn_0.07_Co_0.11_O_2_ (NMC82) exhibit several chemomechanical challenges at the cathode–electrolyte interface, such as contact loss and solid-electrolyte decomposition, resulting in poor interfacial Li^+^ ion transport. To overcome these challenges, we used polymerized ionic liquids (PIL) as coatings at the NMC82 cathode surface, with and without incorporating a lithium salt. The thin Li^+^ ion-conductive Li–PIL nanocoating shows excellent compatibility with sulfide solid electrolytes and enables efficient Li^+^ transfer over the cathode–solid electrolyte interface, as demonstrated by 2D solid-state exchange NMR. It also improves contact retention between the cathode–solid electrolyte particles and mitigates electrolyte oxidation-induced degradation. This is reflected in the electrochemical performance of coated NMC82 in sulfide SSBs, where both a higher rate performance (190 mA h g^−1^*vs.* 163 mA h g^−1^ for uncoated at 0.1C) and a remarkable capacity retention of 82.7% after 500 cycles at 0.2C and ambient conditions (20 °C) are observed. These results emphasize the effectiveness of PILs with Li salts as multifunctional coatings that enable high-performance sulfide-based SSBs with Ni-rich cathode materials at ambient temperature.

## Abstract 2

Li^+^-conducting polymerized ionic liquid coatings chemomechanically stabilize and improve Li^+^ transport at the cathode/electrolyte interface in solid-state batteries.

## Graphical abstract

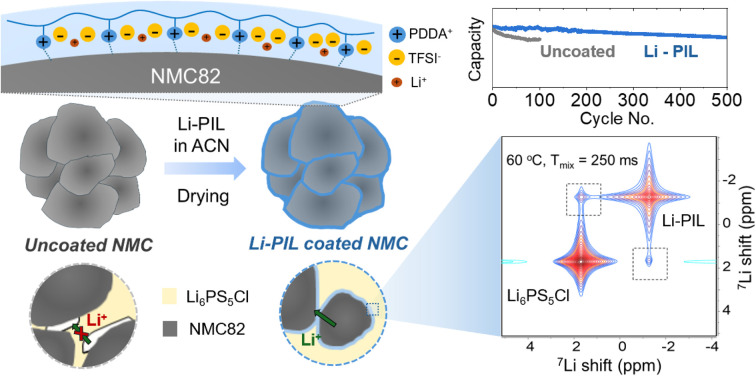

## 1 Introduction

In the last few years, Solid-State Batteries (SSBs) have emerged as a potential electrochemical energy storage technology that could surpass the performance and safety levels of state-of-the-art Lithium-ion Batteries (LIBs).^1,2^ Among the solid electrolytes investigated, sulfides have been receiving widespread attention, owing to the high Li^+^ ionic conductivity at room temperature (>1 mS cm^−1^), coupled with their relative ease of processing and lower gravimetric density compared to other solid electrolytes such as oxides and halides.^2,3^ The cathode is the decisive component in SSBs for battery capacity and energy density.^4,5^ One of the key challenges, therefore, in speeding up the adoption of sulfide-based solid-state batteries is the chemomechanics of the cathode–electrolyte interface.^6,7^ During lithiation and delithiation of the cathode active material (CAM) in all-solid-state-batteries (ASSBs), the resulting volumetric and morphology changes lead to several detrimental effects. Interparticle cracking in polycrystalline CAMs in contact with rigid SE particles creates voids both between the primary CAM particles and at the interface of the CAM and the SE, increasing ionic resistance across the interface.^6,8^ While a move towards single crystal CAMs having a higher primary particle size has been shown to mitigate interparticle cracking,^2,9^ void formation at the CAM/SE interface can still occur due to successive volumetric changes of the CAM, resulting in a loss of surface contact between the SE and the CAM with continuous cycling. One solution that is already widely applied in SSB research is applying high external stack pressure; however, this is considered impractical and does not resolve the issue in its totality.^10^ Furthermore, oxidative decomposition of sulfide-based SEs at the CAM/SE interface leads to the formation of a resistive Cathode Electrolyte Interphase (CEI), leading to irreversible capacity loss.^11^ Oxidation of the widely investigated sulfide electrolyte argyrodite Li_6_PS_5_Cl typically results in species such as polysulfides (Li_*x*_P_*y*_S_*z*_) and elemental sulfur (S^0^).^12^ Furthermore, phosphates (–PO_*x*_) and sulfates (–SO_*x*_) are also reported as oxidation products in combination with LiNi_*x*_Mn_*y*_Co_*z*_O_2_ (NMC hereafter) as the CAM upon extensive cycling. These contribute to a highly resistive CEI with sluggish Li^+^ diffusion.^13^ While inorganic, electronically insulating coatings based on LiNbO_3_,^14^ LiZrO_3_,^15^ LiHfO_2_,^16,17^ and others have been successfully employed to mitigate these processes, application of these coatings typically also results in lowered ionic conductivity at the interface and a lower electronic percolation through the composite, prompting the use of electronic conductive additives in the cathode composite, which could further aggravate the electrochemical instability at the CAM/SE interface.

Therefore, it is desirable to have multifunctional CAM/SE interlayers that possess sufficient (electro)chemical stability and ionic conductivity, which can also buffer volume changes at the CAM/SE interface. In this regard, the CAM particles or the SE could be coated with a polymeric buffer layer.^18^ This polymeric protective coating, when applied on the CAM can inhibit the decomposition reactions of the SE and reduce interfacial resistance between CAM and SE, similar to the inorganic coating. In addition, the polymeric interlayers could also buffer volume change during cycling, thanks to their flexibility.^19^ The polymers used as interlayers can also possess higher ionic conductivity than inorganic coatings, and in some cases, also reasonable electronic conductivity.^18^ Ionically conducting and mixed conducting polymers have already been used as interlayers in both Li-ion batteries and solid-state batteries, resulting in significant performance improvements. Some examples include mixed conducting polymers such as poly(3,4-ethylenedioxythiophene) (PEDOT),^20,21^ PANI–PVP,^22^ cyclized polyacrylonitrile (cPAN),^23^ and single-ion conducting polymers such as lithium polyacrylate (LiPAA) and lithiated sulfonated polyphenylene sulfone (sPPSLi).^24,25^

Polymerized ionic liquids (PILs) present an interesting class of conducting polymers, where the polymeric backbone is either positively or negatively charged, and a counter ion acts as a plasticizer.^26^ Cationic PILs such as poly(diallyldimethylammonium) bis(trifluoromethanesulfonyl)imide (PDDATFSI hereafter) and poly(diallyldimethylammonium) bis(fluoromethanesulfonyl)imide (PDDAFSI) have already found wide application in lithium ion batteries, from being used as solid polymer electrolytes to alternate binders for cathodes.^27–33^ As solid polymer electrolytes, they possess excellent high-voltage stability and show good Li^+^ conductivity and transference at optimized salt concentrations.^29^ In addition, they have been used as interlayers in Li–S batteries, where they have been shown to inhibit polysulfide shuttling, thanks to the charged cationic backbone.^34,35^ Cationic PILs have also been used as interlayers for sulfide-based ASSBs. Shi *et al.* coated PVBA-TFSI on NMC811 using spray drying, and with an optimum loading of 1 wt%, the long-term performance of the ASSBs improved, and this was attributed to the lower buildup of charge transfer resistance at the cathode electrolyte interface.^36^ However, some fundamental questions remain over the role(s) of these coatings. In particular, further light needs to be shed on the potential role of PILs in (electro)chemically stabilizing the CAM/SE interface, and the effect of Li salt addition on Li transport through the coating and over the CAM/SE interface.

In this work, we address the above questions by designing and studying thin polymeric coatings based on PDDATFSI with and without the incorporation of Li salt lithium bis(trifluoromethanesulfonyl)imide (LiTFSI) on commercial Ni-rich LiNi_0.82_Mn_0.07_Co_0.11_O_2_ (NMC82 hereafter). The coatings with and without Li salt, referred to hereafter as Li–PIL and PIL respectively, are characterized to understand their structure, composition, and orientation on NMC82. We also study the ion transport properties in the Li–PIL coating and demonstrate facile Li-ion transport across the Li–PIL coating/SE interface using two-dimensional exchange NMR. The pristine and (Li)PIL coated NMC82 powders are further tested electrochemically in In–Li alloy/Li_6_PS_5_Cl/NMC82 solid-state cells, and the resulting performance is analysed further with detailed post-mortem characterization to elucidate the beneficial effects of (Li)PIL coatings on the cell performance.

## 2 Experimental procedures

The cathode composites were prepared by adding argyrodite Li_6_PS_5_Cl (LPSC, NEI corporation, *D*_50_ = 5 μm), with quasi-single crystal NMC82 (*D*_50_ = 3–5 μm, MSE Supplies LLC) to an agate pestle and mortar in the mass ratio of 30 : 70 and hand-mixing for 15 minutes. No conductive carbon additive was used in the cathode composite. The polymerized ionic liquid of interest, poly(diallyldimethylammonium) bis(trifluoromethanesulfonyl)imide (PDDATFSI, Solvionic), was coated on NMC82 either as a 1 wt% coating without the Li salt, or as a 2 wt% coating with the Li salt, *i.e.* lithium bis(trifluoromethanesulfonyl)imide (LITFSI, Solvionic). Due to the tendency of LiTFSI to absorb moisture from the air, the coating process was performed in an argon-filled glovebox with water oxygen levels below 1 ppm. Either 15 mg of Li–PIL or 7.5 mg of PIL was added to 5 mL of acetonitrile, which was mixed to form a homogeneous solution. To this, 0.75 g of NMC82 was added, and the suspension was mixed for 60 minutes in a closed glass bottle. After 60 minutes, the bottle cap was removed and the suspension was dried at 60 °C, with continued mixing. Finally, the coated powder was dried under vacuum (<10^−3^ mbar) at RT for 72 hours.

The LiTFSI : PDDATFSI (1 : 1 mole ratio) films for NMR characterization were prepared by adding the salt and polymer in the desired ratio to acetonitrile (1 : 1 solvent : solute weight ratio) and mixing until a clear solution was obtained. This solution was spread on a clean polytetrafluoroethylene (PTFE) dish and dried for 12 hours in an Ar filled glovebox, after which the drying was carried out under vacuum (<10^−3^ mbar) at RT for 72 hours to obtain the dried film. A similar procedure was repeated to obtain the hybrid film with Li_6_PS_5_Cl, where the weight of Li_6_PS_5_Cl in the film was set to 30% of the total dry weight of the film.

The cathode composites were tested in custom-made solid-state cells with two stainless steel cell parts surrounded by a PEEK sleeve with a cavity of 10 mm (assembly details are shown in Fig. S1†). First, 60 mg of LPSC was compressed at 125 MPa to form the separator. Cathode composites corresponding to 10 mg cm^−2^ (≈2 mA h cm^−2^) of CAM loading were added to one side of the separator, and pressed at 312.5 MPa. Finally, the InLi alloy foil was prepared by placing an In foil with 8 mm diameter on the LPSC separator, followed by a Li foil with 5 mm diameter on top. The weight ratio of indium to lithium was optimized as per the work of Santhosha *et al.*^37^ The final cell was closed at 60 MPa pressure.

The galvanostatic cycling of the batteries was performed on MACCOR 4300 cycler at 20 °C. The solid-state batteries were charged and discharged between 2.15 V and 3.7 V (corresponding to 4.3 V *vs.* Li^+^/Li). Two initial cycles were run at a C-rate of 0.05C (0.1 mA cm^−2^), followed by further cycling at 0.2C (0.4 mA cm^−2^). A minimum of two cells were tested for each NMC82 powder type. Electrochemical impedance spectroscopy measurements were performed on AUTOLAB potentiostat (Metrohm Inc.) at 20 °C, after the (two) initial cycles, and after 100 cycles. The cells were charged to an open circuit voltage (OCV) of 3.0 V (3.62 V *vs.* Li^+^/Li). Measurements were taken between 10 MHz and 0.1 Hz. Further processing and analysis of the spectra were performed using RelaxIS 3 software (RHD Instruments). Distribution of Relaxation Times (DRT) analysis was conducted, also using RelaxIS 3, to serve as an aid in the fitting of equivalent circuits to the impedance data. Bruce–Vincent Li^+^ transference number measurement at 60 °C was also performed on the AUTOLAB potentiostat in Li/Li symmetric cells with a 250 μm Li–PIL film as the separator. A DC amplitude of 5 mV was used for the potentiostatic polarization until steady-state conditions were reached.^38^

XPS measurements were performed on NMC82 powders before and after the coating, and on the cathode composites before and after cycling. For the post-cycling (100 cycles) XPS measurements, the samples were retrieved from cells after equilibration at an OCV of 3.0 V (3.62 V *vs.* Li^+^/Li). The samples are handled in the glove box and placed into a vacuum transfer holder before being placed in the XPS apparatus. The XPS measurements were performed on a Thermo Scientific K-Alpha Spectrometer with an Al K-alpha monochromator. The spot diameter was set to 400 μm. Survey spectra were recorded with a pass energy of 200 eV and step size of 0.5 eV, while high-resolution spectra were recorded with a pass energy of 50 eV and step size of 0.1 eV. The binding energies were referenced to adventitious carbon at 284.8 eV. All the spectra were processed and analysed using the CasaXPS software using a Shirley background to account for inelastically scattered photoelectrons.

Scanning Electron Microscopy (SEM) measurements were performed on JEOL JSM-IT100 for samples in an airtight holder, and JEOL JSMIT700HR FE-SEM for NMC powder investigations. For the investigation of cathode composites, Backscattered Electron Detection (BED) images were taken with electron acceleration voltages of 10–15 kV to obtain sufficient contrast between Li_6_PS_5_Cl, NMC82, and voids. For the investigation of polymer coatings on NMC82, low electron acceleration voltages of 1 keV were used in the Secondary Electron Detection (SED) mode. Energy Dispersive X-ray Spectroscopy (EDS) point analysis and elemental mapping were carried out on coated NMC82 with an electron acceleration voltage of 5 kV.

The mechanical compression tests were carried out on a TA RSA G2 solids analyser using 15 mm disc compression plates. A ∼600 μm Li–PIL film was subjected to uniaxial compression of up to 100 μm at a rate of 2 μm s^−1^, followed by recovery at the same rate.

X-ray Diffraction (XRD) measurements were performed on the X'Pert Pro X-ray diffractometer (PANalytical). Cu Kα X-rays (1.5406 Å at 45 kV and 40 mA) were used, with the absolute scan function and the XCelerator detector. The samples were tested in airtight holders with a Kapton window. Diffraction patterns were collected in a 2*θ* angular range from 10° to 90°, with a step size of 0.008°. Pawley refinement and fitting of the resulting spectra was performed using TOPAS software (Bruker).

For Transmission Electron Microscopy (TEM) measurements, a Thermo Fisher Scientific Titan Cs-corrected 80–300 kV TEM was used. The coated powders were gently crushed and put into hexane and the suspension was ultrasonically shaken for 2 minutes, and 2 droplets were loaded onto a TEM grid with a holey carbon foil. Elemental mapping in STEM mode was done, using the Super-X in the ChemiSTEM™ configuration. In STEM mode, a small electron beam (∼0.3 nm) scanned the specimen. For each beam position, the diffracted electrons were collected on a ring detector, thus forming a High Angle Annular Dark Field (HAADF) image after the complete area was scanned. At the same time, an EDX spectrum was collected for each beam position, and elemental maps were made. Two-pixel averaging was applied.

All solid-state NMR measurements were carried out on a Bruker Ascend 500 MHz (11.7 T) spectrometer equipped with a Neo console. For the Li–PIL polymer and the coated NMC82 powder, measurements were carried out on a 3.2 mm triple resonance probe at spinning speeds of 15–20 kHz, while for the cathode composites (pre and post-cycling), the measurements were performed on 1.9 mm double resonance probes at 35 kHz spinning speed. ^19^F and ^1^H MAS NMR measurements were acquired using a rotor-synchronized Hahn echo protocol, while ^13^C measurements were performed with direct excitation pulses with high power (30–60 W) proton decoupling. For ^13^C measurements, a pulse length of 3.1 μs (145 W) was used with a recycle delay of 1–2 s. ^1^H measurements were carried out with a pulse length of 2.8 μs (80 W) and recycle delay of 1–5 s. ^1^H and ^13^C measurements were referenced to adamantane at 1.77 ppm and 37.5 ppm respectively. ^19^F measurements were carried out with a pulse length of 2.8 μs (80 W) and a recycle delay of 1–10 s. ^1^H → ^13^C CP MAS experiments were performed at 10 kHz with an initial ^1^H π/2 pulse of 2.8 μs and contact times ranging from 0.05 to 2 ms. A radio frequency field strength of 64 kHz was utilized, with a recycle delay of 2 s. The field amplitude of ^1^H was ramped from 70 to 100% and a TPPM15 sequence was used for proton decoupling. ^7^Li–^7^Li 2D EXSY NMR measurements were performed at 60 °C with mixing times ranging from 10 ms to 250 ms and a recycle delay of 0.4 s. The 2D spectra consisted of 32 scans each for 3000 transients, with each transient incremented by 514.6 μs. The processing and analysis of the resulting spectra was performed using the Mestrenova 14 software.

For PFG NMR, a Bruker Ascend 600 (*B*_0_ = 14.1 T) magnet equipped with a NEO console was used. The samples were measured using stimulated echo pulse field gradient procedure on ^7^Li (π/2 pulse length of 17.5 μs, 45 W and *B*_1_ = 80–1600 gauss per cm) and ^19^F (π/2 pulse length of 23 μs, 15.5 W and *B*_1_ = 80–1600 gauss per cm) using a linear gradient of 8 slices with typical diffusion times of 100–400 ms, gradient pulse durations of 1–2.5 ms, and 8–64 scans per slice for temperatures from 35 °C to 75 °C for every 5–10° increment in temperature. The data was fit using the Stejskal–Tanner equation, and Bruker Dynamics Center software was used.

## 3 Results and discussion

### 3.1 Materials selection and design for polymeric coatings on Ni-rich CAMs

An important criterion to be fulfilled by polymeric surface coatings for Ni-rich CAMs is high oxidative (anodic) stability. In this regard, the polymer investigated here, *i.e.* PDDATFSI, has previously shown high anodic stability of up to 5 V *vs.* Li^+^/Li,^27^ and this can be attributed to the presence of cyclic quaternary ammonium cations in its polymeric backbone and –CF_3_ moieties.^39^ We limited the polymer loading on the cathode surface to 1–2 wt% of the cathode active mass, as non-lithiated PIL coatings with similar mass loading ranges were shown to result in optimal long-term cycling for sulfide SSBs.^36^ For Li salt containing PILs, the optimization of salt concentration is also necessary to balance ionic conductivity and transference while avoiding local salt crystallization. Previously, PIL repeat unit : salt ratio (mol%) of 1 : 1.5 resulted in optimal performance for LiFSI : PDDAFSI and LiTFSI : PDDATFSI based systems.^29,30^ In our case, a salt ratio of 1 : 1 (mol%) was chosen, given the high surface-to-volume ratios expected for thin (nanometer range) coatings and possible confinement effects in this range, leading to lower solubility and undesirable salt precipitation.^40^

**Fig. 1:**
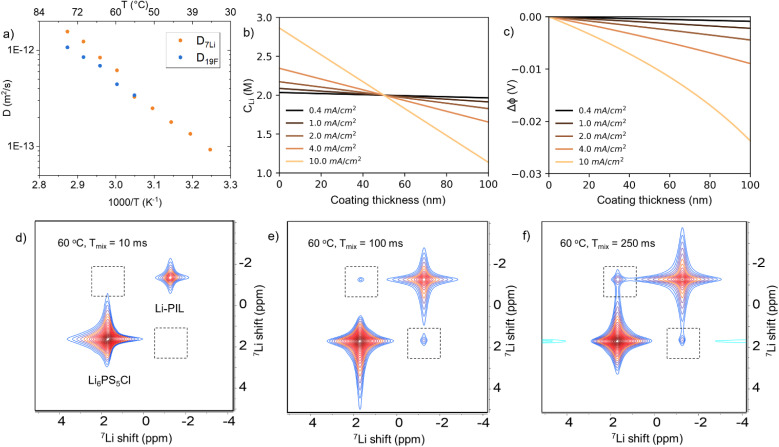
(a) ^7^Li and ^19^F PFG NMR diffusivities of 1 : 1 Li–PDDATFSI PIL (Li–PIL) as a function of temperature. (b) Concentration profiles and (c) potential drops after 2 mA h cm^−2^ of electrochemical cycling at different current densities across a 100 nm Li–PIL coating. Bottom: ^7^Li–^7^Li 2D EXSY NMR spectra at 60 °C performed on Li–PIL – 30 wt% Li_6_PS_5_Cl hybrid electrolyte films with mixing times of (d) 10 ms, (e) 100 ms and (f) 250 ms.

For the chosen salt concentration, the ^7^Li and ^19^F PFG NMR results of Li–PIL performed at a temperature range of 35–75 °C (Fig. 1a) show a high Li* diffusivity of 9.27 × 10^−14^ to 1.56 × 10^−12^ m^2^ s^−1^ and F* diffusivity of 3.43 × 10^−13^ to 1.07 × 10^−12^ m^2^ s^−1^ (55–75 °C) respectively. When extrapolated to 20 °C, the Li* diffusivity obtained is around 3.3 × 10^−14^ m^2^ s^−1^, which is higher than that of most inorganic coatings typically applied on Ni-rich CAMs.^41^ The Li* transport numbers (ratio of Li* diffusivity to the total diffusivity) based on these diffusivities are also high, *i.e.* 0.49–0.59 for the probed temperature range (Fig. S2†). The electrochemically measured ionic conductivity ranges from 5.02 × 10^−7^ S cm^−1^ at 20 °C to 3.19 × 10^−5^ S cm^−1^ at 70 °C (Fig. S3a†), while the Bruce–Vincent Li^+^ transference number is about 0.48 at 60 °C (Fig. S3b†). While PILs are known to have a significant degree of ion–ion correlations, resulting in practically much lower electrochemical conductivities than those indicated by self-diffusivities,^42^ the inverse Haven ratio, *i.e.* the ratio of electrochemically measured conductivity to the self-diffusivity-based, uncorrelated conductivity from PFG-NMR, calculated at 60–70 °C is about 0.19–0.23 for Li–PIL (more details provided under ESI Note 1†). These values are much higher than those typically reported for solid polymer PILs (0.05–0.125) and comparable to that of gel polymer PILs (∼0.2) at these temperatures,^42,43^ indicating the presence of Li^+^ ions in PIL with a 1 : 1 molar ratio significantly improves the free ion mobility in the system. Overall, our results highlight the favourable Li^+^ ion transport properties for the chosen Li–PIL composition over a wide temperature range.

Consequently, the use of Li–PIL results in minimal potential drops (of up to 0.03 V) and no concentration polarization across the coating layer after 2 mA h cm^−2^ of electrochemical cycling at 20 °C for current densities up to 10 mA cm^−2^ and coating thicknesses of up to 100 nm, as shown by the analytical solution results for the Nernst–Planck transport equation (Fig. 1b and c, methodology further described under ESI Note 1†).^44^ This is despite our assumption of a conservative lower bound of 10^−15^ m^2^ s^−1^ for Li^+^ diffusivity, to account for changes in ion transport properties due to possible ion confinement effects at low coating thicknesses and the aforementioned ion–ion correlations at these concentrations.^29,40,45^ These results suggest that the Li–PIL, with its good balance of Li^+^ conductivity and transference, presents a competitive alternative to other commonly probed polymer (electrolyte) materials for application as surface coatings in SSBs operated under ambient temperature conditions.

It is to be noted that adequate Li^+^ transport properties in the bulk polymer electrolyte do not always translate into sufficient Li^+^ transport properties across the polymer/SE interface, as previously observed for PEO–LiTFSI systems, where the nucleophilic attack-induced decomposition products formed between PEO and Li_6_PS_5_Cl impede interfacial transport.^46,47^ However, the Li–PIL coating, with its less nucleophilic, positively charged polymer backbone and higher (electro)chemical stability, is expected to contribute positively to Li^+^ transport across the CAM/SE interface. 2D exchange (EXSY) ^7^Li NMR, previously employed to probe Li^+^ ion exchange across different chemical environments including Li_6_PS_5_Cl/electrode and Li_6_PS_5_Cl/polymer interfaces,^46,48–50^ was employed to provide confirmation of Li^+^ transfer over the Li–PIL/LPSC interface. These measurements were performed at 60 °C on Li_6_PS_5_Cl/Li–PIL hybrid solid electrolytes (with 30 wt% Li_6_PS_5_Cl). Here, the mixing time, *i.e.* Li self-diffusion period, is varied between 10 ms and 250 ms to probe the typical time scales of Li transport over the Li_6_PS_5_Cl/Li–PIL interface (Fig. 1d–f). These mixing times are short enough compared to the *T*_1_ relaxation time of Li_6_PS_5_Cl (∼500 ms) but long enough to show Li diffusion between Li_6_PS_5_Cl and Li–PIL, *i.e.* diffuse over similar 10–20 nm thicknesses of Li–PIL coating layers.

With a short mixing time of 10 ms, cross peaks, which correspond to Li^+^ exchange over the LPSC/PIL interface do not yet appear (Fig. 1d). However, they are observed with a longer mixing time of 100 ms and further intensify with a longer mixing time of 250 ms (Fig. 1e and f). This is in sharp contrast to PEO–LiTFSI/Li_6_PS_5_Cl systems, where no Li^+^ exchange was observed even after 2000 ms of mixing time at 55 °C.^46^ These results indicate that the addition of Li salt to the PDDATFSI coating results in the participation of the PIL phase in Li^+^ conduction and facile Li^+^ transfer over the Li–PIL/LPSC interface.

### 3.2 Structural and chemical characterization

**Fig. 2:**
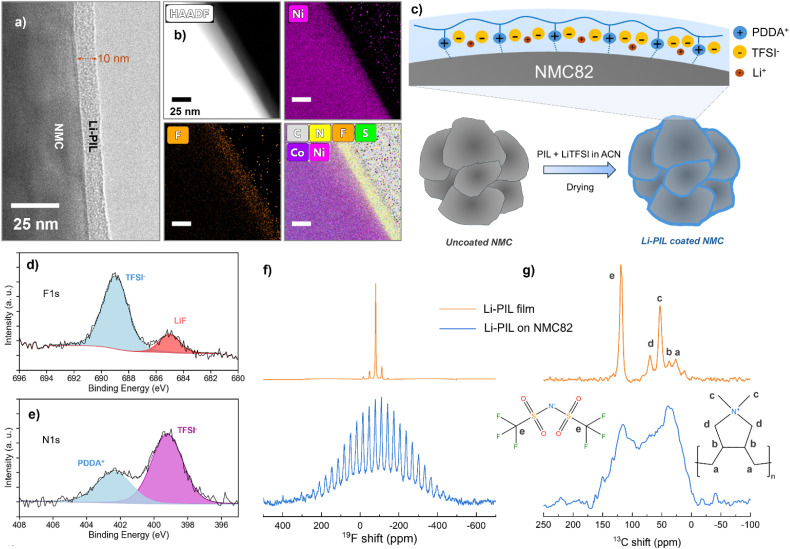
Characterization of PIL coatings on NMC82: (a) TEM image of 2 wt% Li–PIL coating on NMC82 (b) HAADF, and STEM elemental at% mapping of Ni, F, S and N, for Li–PIL on NMC82. (c) Schematic diagram depicting the changes to the NMC82 surface before and after coating with Li–PIL (d) high-resolution F 1s and (e) high-resolution N 1s XPS spectra of NMC82 powder coated with Li–PIL. (f) ^19^F Hahn echo MAS NMR spectra of Li–PIL film (top) and NMC82 coated with Li–PIL (bottom). (g) ^13^C proton decoupled (60 W) MAS NMR spectra of Li–PIL film (top) and NMC82 coated with Li–PIL (bottom). All measurements were performed at 15–20 kHz spinning speeds.

The 2 wt% Li–PIL and 1 wt% PIL coatings deposited on the surface of NMC82 were analysed to determine their nature, composition, and possible effects on the NMC crystal structure, and the results are summarized in Fig. 2. Transmission Electron Microscopy (TEM) was performed using a Cs-corrected Thermo Fisher Scientific Titan operated at 300 kV equipped with a Super-X EDX detector in the ChemiSTEM™ configuration, with a focus on determining the thickness, morphology, and elemental distribution of the PIL coating. The surface of the NMC82 particle is shown to be covered by a PIL coating, with a thickness of ∼10 nm (Fig. 2a). The High Angle Annular Dark Field Scanning Transmission Electron Microscopy (HAADF-STEM) and the Energy Dispersive X-ray spectroscopy (EDX) atomic percentage (at%) map images (Fig. 2b) reveal a thin, uniform distribution of F, S, C and N elements on the particle surface with Ni and Co present in the bulk (Fig. 2b and S5†). It is to be noted, as observed from the Scanning Electron Microscopy (SEM) images (Fig. S6†), that the commercial NMC82 consists of agglomerates of particles, and this structure is retained after the coating. The TEM images of coated NMC82 agglomerate (Fig. S7,† zoomed images indicating the coating thickness shown for different regions A–F) indicate that the outer surfaces of the agglomerate are coated with a thickness of ∼5–25 nm. The Li–PIL coating was also observed to deposit between the NMC82 grains, in this case with a higher thickness (Fig. S8†). Furthermore, the Energy Dispersive X-ray Spectroscopy (EDS) point analysis and elemental mapping carried out over multiple coated NMC82 particles (Fig. S9†) minimal polymer aggregation over the coated surfaces. The employed wet coating method is, therefore, expected to coat mainly the outer surfaces of the agglomerate with a higher coating thickness between the primary particles (Fig. 2c).

To further elucidate the chemical environment of the observed coating in comparison to that of the bare NMC82 powder, we performed X-ray Photoelectron Spectroscopy (XPS) measurements on the coated and uncoated NMC82 powders (Fig. 2d, e and S10†). The F 1s spectrum of Li–PIL coated NMC82 (Fig. 2d) shows a main peak at ≈689 eV corresponding to the TFSI^−^, and a secondary peak corresponding to LiF (≈685 eV), which could be present due to LiTFSI degradation under X-rays.^51^ In the N 1s spectrum (Fig. 2e), the peak at ≈402.5 eV corresponds to the quaternary amine from PDDA^+^, while the peak at ≈399 eV corresponds to nitrogen in the TFSI^−^.^52^ Furthermore, the C 1s spectrum shows an increased presence of C–N and C–F groups for the Li–PIL coated powders, and these findings are further corroborated by the elemental atomic percentages from the survey spectra of the uncoated and coated powders (Fig. S11†).

Magic angle spinning (MAS) solid-state NMR results provide further insights into the uniformity of Li–PIL coating and its possible orientation on the NMC82 surface. ^1^H, ^19^F, and ^13^C spectra of Li–PIL coated NMC82 are compared with those of the Li–PIL film (LiTFSI/PDDATFSI (1 : 1 mol%) solid polymer electrolyte film prepared using wet coating/drying (with acetonitrile as the solvent)). For the Li–PIL coated NMC82, a broad background of ∼800 ppm is observed for the ^19^F Hahn echo spectrum (Fig. 2f), while the spinning sideband manifold also has a similar range and is wider in comparison to that of the PIL film. Moreover, the diamagnetic peak corresponding to TFSI^−^ at ∼−80 ppm is broader for the coated powder. A similar phenomenon is observed for ^1^H Hahn echo NMR; a much broader background signal is observed for the Li–PIL coated NMC82 (Fig. S12†), and this feature is absent for the Li–PIL film. For the ^13^C spectra with high power (60 W) proton decoupling (Fig. 2g), five distinct peaks are observed for the Li–PIL film, with the peaks at ∼52.5 ppm (corresponding to the CH_3_ groups dangling from N^+^) and ∼125 ppm (corresponding to TFSI^−^) being the most intense, but this changes for the coated powders. Instead of sharp peaks, a broad manifold is observed, with the highest intensity for the region corresponding to the PDDA environments occurring close to 25–40 ppm. The TFSI^−^ peak at ∼125 ppm also appears broader. The lineshape broadening of the PDDA carbons on coated powders is also further confirmed with ^1^H–^13^C cross-polarization (Fig. S13a†) where a broad manifold is again observed instead of individual peaks for the coated powders. The broad background observed for ^1^H and ^19^F, and the broader lineshapes observed for ^19^F and ^13^C can both be attributed to the influence of the paramagnetic interactions with the NMC82 core. This implies that most of the coating applied on NMC82 is in sufficient proximity (few nanometers) to be influenced by the paramagnetism of NMC82, which further corroborates the TEM results.

It is interesting to note the change in the relative intensities for the ^13^C environments going from Li–PIL film to the coated NMC82 powder, which could offer insights into the orientation of the Li–PIL coating on NMC82 surface. To further study this phenomenon, ^13^C cross-polarization dynamics NMR (sensitive to polymer dynamics) experiments were performed on the Li–PIL film and 2 wt% Li–PIL coated on non-paramagnetic LiCoO_2_ (Fig. S13b and c†) to exclude the effects of paramagnetic line broadening. Here, the results show that the intensity for the –CH_3_ groups directly bonded to N (52.5 ppm) stays nearly the same after a small increase, and does not decrease with increasing contact times for cross-polarization from 0.05 to 1 ms, indicating a relative decrease in mobility for this environment (Fig. S13d and e†).^53^ This could mean that the Li–PIL coating has the positively charged polymer backbone (with the quaternary N^+^) drawn towards the NMC82 surface (Fig. 2c). This could be due to the electrostatic attractive forces induced by the negatively charged outer surface, which can be expected for layered oxides like NMC82. For the effect to be discernible by NMR, it is required that such electrostatic interaction propagates throughout the Li–PIL layer. If so, this could be a part of the explanation why the NMC82 particles can be successfully coated with Li–PIL, and it can be argued that cationic polymers hold an intrinsic advantage over anionic and neutral polymers for applications as coatings on layered oxides. However, further experiments would be necessary to establish this hypothesis.

We also investigated the structural stability of NMC82 before and after coating with X-ray Diffraction (XRD) (Fig. S14†). The characteristic peaks (003), (101), and (110) at around 18.8°, 36.6°, and 64.8° respectively, corresponding to the *R*3̄*m* space group of NMC82, were used to study the evolution of the lattice parameters *a*, *b*, and *c*.^54^ It is observed that the calculated lattice parameters (Fig. S14,† insets) stay nearly the same before and after coating (2.8726 Å *vs.* 2.8732 Å for *a*, *b*; 14.1909 Å *vs.* 14.1917 Å for *c*). This implies that the NMC82 retains its original crystal structure and particle size after the coating. Moreover, no additional environments corresponding to crystalline LiTFSI were observed in the spectrum, implying that the chosen salt ratio for the coating does not result in localized salt precipitation when coated on NMC82.

Together, the above results suggest that Li–PIL has been deposited on the surface of NMC82 particles as a thin nanometric coating with the intended chemical composition, and no changes to the NMC82 crystal structure.

### 3.3 Electrochemical cycling performance

**Fig. 3:**
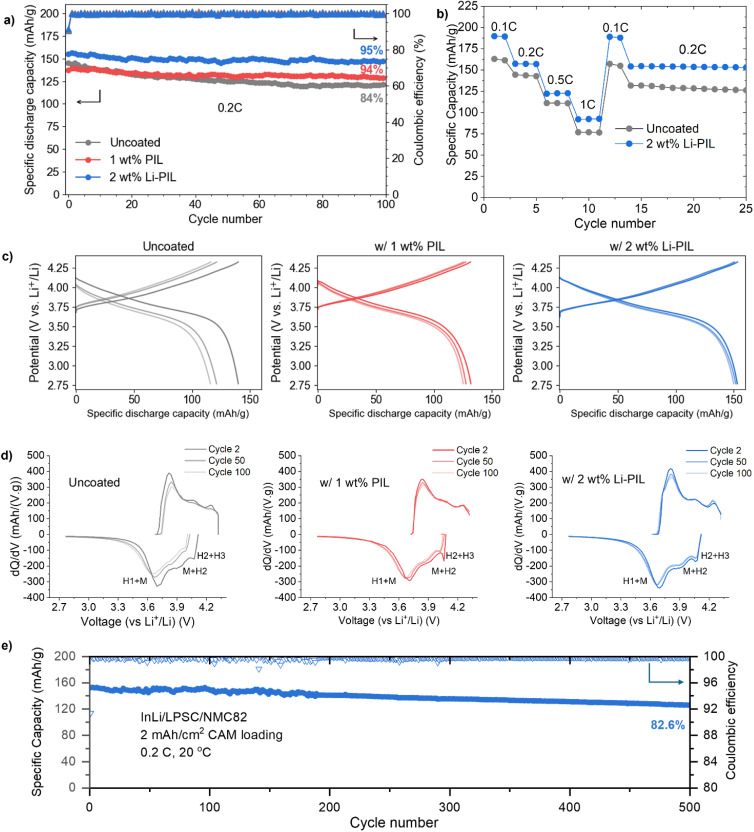
Electrochemical performance comparison: (a) discharge capacities and coulombic efficiencies for 100 cycles of InLi/Li_6_PS_5_Cl/NMC82 cells for uncoated NMC, NMC with PIL, and NMC with Li–PIL, at 0.2C and 20 °C, and active mass loading of 10 mg cm^−2^ (b) charge–discharge voltage profiles for the uncoated, PIL and Li–PIL cells at 0.2C for the 2nd, 50th, and the 100th cycle. (c) First cycle coulombic efficiencies for the uncoated, PIL and Li–PIL cells at 0.05C. (d) Rate performance comparison for the uncoated and Li–PIL cells. (e) Long-term discharge capacity and coulombic efficiency of InLi/Li_6_PS_5_Cl/NMC82 cells for NMC with Li–PIL, at 0.2C and 20 °C, and NMC82 loading of 10 mg cm^−2^ for 500 cycles.

To investigate the impact of (Li)PIL coatings on the rate capability and the long-term performance of solid-state batteries, we performed galvanostatic cycling of coated and uncoated NMC82 in InLi/Li_6_PS_5_Cl/NMC82 cells. The long-term cycling performance of these solid-state cells is compared over 100 cycles at 0.2C and at 20 °C (Fig. 3a). Here, the initial discharge capacity of Li–PIL coated NMC82 is the highest at 155.2 mA h g^−1^, followed by the pristine NMC82 at 144.9 mA h g^−1^ and then the PIL-coated NMC82 at 137.3 mA h g^−1^. After 100 cycles, the capacity retention of the sample with Li–PIL and PIL are similar at about 94%, and 95%, respectively. However, the Li–PIL coating also improves the initial capacity, whereas the PIL coating reduces the initial capacity. The capacity retention of the pristine NMC82 is only ≈84%.

The improvement in initial capacity for the Li–PIL coated NMC82 is also reflected in the rate performance tests performed for C-rates from 0.1C up to 1C (Fig. 3b). At all C-rates, the Li–PIL coated NMC82 outperforms the uncoated NMC82 (190 mA h g^−1^*vs.* 163 mA h g^−1^ at 0.1C, 92.7 mA h g^−1^*vs.* 76.6 mA h g^−1^ at 1C). On returning to lower C-rates, the capacity retention is observed to be much better (157.3 mA h g^−1^ to 154.5 mA h g^−1^ at 0.2C *vs.* 143.6 mA h g^−1^ to 131.8 mA h g^−1^ at 0.2C) for the Li–PIL coated NMC82.

The PIL and the Li–PIL coatings significantly improve the capacity retention of NMC82. Fig. 3c shows the charge and discharge curves for the 2nd, 50th and 100th cycle of the three samples. For the sample with pristine NMC82, most of the capacity deterioration occurs in the first 50 cycles. With polymeric protective coatings PIL and Li–PIL, a lower capacity fade can be detected for the first 50 cycles, slowly heading towards a stable system with negligible capacity fade over the last 50 cycles. This is also reflected by the d*Q*/d*V* plots for the three systems (Fig. 3d), where a significant reduction of the reversibility in M to H2 and H2 to H3 phase transitions is observed for uncoated NMC82, whereas the Li–PIL coated NMC82 powder shows high reversibility of these transitions at cycle 100 (Fig. 3d). Furthermore, no additional peaks are observed for the (Li)PIL d*Q*/d*V* plots, implying that there are no obvious side reactions involving these polymer coatings during cycling.

On the charge transfer kinetics, an overlay comparison of voltage traces at cycle 2 and cycle 100 (Fig. S15†) reveals that in the case of the 1 wt% PIL, the discharge voltage trace of cycle 2 shows a steeper slope towards the end of discharge, which could be due to sluggish charge transfer across the PIL layer. In contrast, the 2 wt% Li–PIL sample has the same slope as the uncoated sample, but with higher overall discharge, which suggests an increase in CAM/SE interfacial area without any charge transfer limitations. After 100 cycles, however, the uncoated sample displays a more sluggish charge transfer kinetics, while the 1 wt% PIL and 2 wt% Li–PIL retain nearly the same slope for the voltage profiles.

The application of coatings on NMC82 also improves the first cycle coulombic efficiency (Fig. S16†), which serves as an indicator of the extent of side reactions such as SE oxidation during the initial cycling.^17^ Here, while the PIL coating results in an improvement of the 1st cycle CE from 77.3% to 78.7%, the Li–PIL results in further improvement to 79.5%. While this suggests that the PIL coating contributes towards improved (electro)chemical stability with Li_6_PS_5_Cl, the additional improvement in CE for the Li–PIL coated NMC82 could be due to the improved Li^+^ transfer kinetics during discharge. The Li–PIL coating improves both the rate performance and long-term stability of the LPSC/NMC system, and this is reflected in the excellent capacity retention of 82.7% at 0.2C and 20 °C for cells with 10 mg cm^−2^ (∼2 mA h cm^−2^) CAM loading after 500 cycles (Fig. 3e). Table S2† compares the performance of Li–PIL coated NMC82 with previous reports on Ni-rich NMCs with organic/inorganic coatings in sulfide SSBs, demonstrating that our coating strategy is highly competitive in terms of both initial capacity and long-term retention.

Electrochemical Impedance Spectroscopy (EIS) results provide insights into the differences in resistance evolution at the Li_6_PS_5_Cl/NMC82 interfaces for the coated and uncoated powders.

**Fig. 4:**
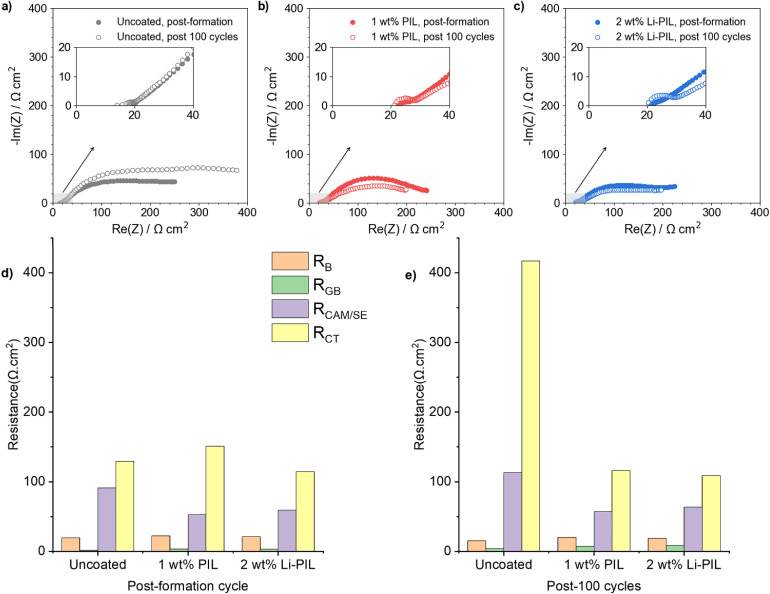
EIS comparison: (a) EIS Nyquist plots for uncoated NMC82, (b) NMC82 coated with PIL, and (c) NMC82 coated with Li–PIL, both post-formation cycles and post-100th cycle. All measurements were taken at 20 °C and at 3 V (3.62 V *vs.* Li^+^/Li) OCV. (d) Resistances *R*_B_, *R*_Sep_, and *R*_CAM/SE_, post-formation, for the samples NMC82, NMC82 with PIL, and NMC82 with Li–PIL. (e) Resistances *R*_B_, *R*_Sep_, and *R*_CAM/SE_, post-100 cycles, for the samples NMC82, NMC82 with PIL, and NMC82 with Li–PIL. The equivalent circuit and the resulting fits are shown in Fig. S17.†

Fig. 4a–c show the Nyquist plots of the samples NMC82, NMC82 coated with PIL, and NMC82 coated with Li–PIL, post-formation cycles and post-100 cycles. All EIS measurements were performed after equilibration at an OCV of ≈3.0 V (or 3.62 V *vs.* Li^+^/Li) to maintain a similar state of lithiation in the cathode composites. Given the similar range of time constants for different electrochemical processes in SSBs resulting in convoluted Nyquist plots, we also carried out Distribution of Relaxation Times (DRT) analysis to better deconvolute the individual processes and find an equivalent circuit.^55^ The DRT analysis, the equivalent circuit for fitting the EIS measurements and the resulting fits are shown in Fig. S17.†

The resistances *R*_B_, *R*_GB_, *R*_CAM/SE_ and *R*_CT_, determined using the fitted impedance data, are shown in Fig. 4d and e (and values are provided in Table S3†). *R*_B_ corresponds to the bulk SE (Li_6_PS_5_Cl) resistance, *R*_GB_ refers to the grain boundary resistance in the argyrodite separator, *R*_CAM/SE_ refers to the mechanical contact resistance between the CAM/SE phases,^56^ and *R*_CT_ refers to the charge transfer resistance at the cathode particle interface (overlapping with the anode charge transfer resistance).^56,57^ The uncoated NMC82 shows the highest *R*_CAM/SE_ value initially, and this could be due to the relatively poor mechanical contact between CAM/SE phases in the absence of a coating. Post-cycling, the interface resistances for the uncoated NMC82 increase significantly, where *R*_CT_ is nearly 4 times its value pre-cycling, and *R*_CAM/SE_ also increases. This is in line with the capacity fade the cell displayed during electrochemical cycling (Fig. 3a). The significant increase in *R*_CT_ could be caused by the formation of a resistive CEI due to Li_6_PS_5_Cl decomposition, and the increase in *R*_CAM/SE_ indicates increased contact loss between the CAM/SE particles in the composite.

For the NMC82 coated with 1 wt% PIL, the coating could improve the CAM/SE contact area and offer mechanical stability to this interface during cycling, which could explain the low *R*_CAM/SE_, both before and after 100 cycles. However, the initial absence of Li^+^ in the coating could result in a high activation barrier for Li^+^ transfer, leading to sluggish transport (Fig. S15†). PDDATFSI, when used as a binder in Li–S batteries, was postulated to allow some Li^+^ hopping through weakly associated and mobile TFSI^−^ counterions of the polymer, and the availability of Li^+^ ions at the interface gradually improved with cycling due to the formation of LiTFSI at the interface through the anion metathesis reaction involving polysulfides trapped in the coating.^34^ While such trapping and the additional Li^+^ exchange step could also be initially contributing to the heightened kinetic barrier to Li^+^ transfer, resulting in a high *R*_CT_, a gradual increase in LiTFSI concentration at the interface with cycling would lower this kinetic barrier, which can also explain the lowered *R*_CT_ after 100 cycles.

In the case of 2 wt% Li–PIL, while the interfacial mechanical stability improves, similar to the 1 wt% PIL layer, the presence of significantly higher Li^+^ ions in the coating also lowers the kinetic barrier to Li^+^ ion transfer across the interface. This results in both low *R*_CAM/SE_ and *R*_CT_ values before and after 100 cycles, which agrees well with the observed improvements in both rate capability and cycle life.

While some differences are observed in *R*_B_ and *R*_GB_ values of the cells both before and after 100 cycles, we attribute these to differences arising from cell-to-cell variations in assembly and solid-state separator microstructure, reported by several SSB research laboratories as part of a recent round-robin study.^58^ Furthermore, these differences are negligible compared to the much higher differences observed for *R*_CAM/SE_ and *R*_CT_.

### 3.4 Effect on (electro)chemical stability

**Fig. 5:**
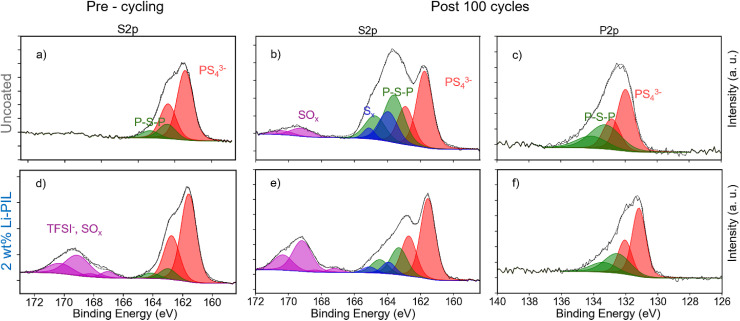
Pre-cycling S 2p and post-cycling S 2p and P 2p XPS spectra of (a–c) uncoated NMC82 and (d–f) NMC82 with Li–PIL coating.

Li–PIL, with its high oxidative stability and interfacial compatibility with Li_6_PS_5_Cl, the latter demonstrated by 2D EXSY NMR (Fig. 1d–f), is expected to alleviate the electrochemical degradation of the CAM/SE interface. To investigate this further, we performed XPS measurements on the cathode composites before and after cycling. The pre-cycling S 2p and post-cycling S 2p and P 2p spectra are shown for the coated and uncoated samples in Fig. 5.

For the S 2p spectra (Fig. 5a, b, d and e), four different peak doublets are observed. The main component is detected at a binding energy of ≈161.7 eV, corresponding to the PS_4_^3−^ units of the Li_6_PS_5_Cl.^59^ Oxidized species were detected at roughly 162.9 eV and 163.9 eV corresponding to the P–S–P (P_2_S_*x*_) and S_*x*_, respectively. P–S–P – or polysulfides, are known to form during the mixing of NMC with Li_6_PS_5_Cl.^60^ S_*x*_ here could indicate multiple compounds, including elemental sulfur that can be responsible for the intensity at ≈163.9 eV.^61^ In the case of the coated samples, an additional environment is observed at ≈169.4 eV corresponding to TFSI^−^.

For both composite cathodes, electrochemical cycling increased the intensities of oxidized species in the S 2p spectra (Fig. 5b and e). However, in the case of uncoated NMC, the intensity of the oxidized species was greater than for the samples coated with Li–PIL. Additionally, oxidized sulfates (likely SO_4_^2−^) were detected in the uncoated sample at ≈169.0 eV. However, the intensity at the same binding energy for coated samples corresponds to the units of TFSI^−^ and therefore, it is hard to visually determine if these SO_*x*_ compounds were also formed in the case of the coated powders with cycling. However, a comparison of the area-wise percentages for the S 2p components provided in Fig. S18† indicates that the TFSI^−^/SO_*x*_ environment stays nearly the same with cycling. This is also reflected in the ratios of the TFSI^−^ environments in the F 1s and S 2p spectra remaining nearly the same with cycling (Table S4†). It is also to be noted that Li_6_PS_5_Cl is known to display some degree of reversible redox upon charging and discharging,^12^ and some part of the observed oxidized species might indeed be reversible. However, all the cells for post-mortem analysis were disassembled at an OCV of 3 V (3.62 V *vs.* Li^+^/Li) to ensure a uniform state of charge across these samples. Therefore, the reduction in the amount of P–S–P and S_*x*_ species post-cycling is evident for the powders coated with Li–PIL.

For the P 2p spectra (Fig. 5c and f), a similar trend is observed, with PS_4_^3−^ at around 131 eV and polysulfides at approximately 132.5 eV. A similar ratio of PS_4_^3−^ to P–S–P was detected as in the S 2p spectra for all cells. The average signal position for the polysulfides shows slight deviations per cell, which is a result of the average chain length of the number of sulfide atoms, P–[S]_*n*_–P, in the polysulfides.^60^ A signal shift towards higher binding energies with increasing *n* is assumed, which is observed in the uncoated NMC82 composite (Fig. 5c). Again, the relative fraction of oxidized P–[S]_*n*_–P species is substantially higher for the uncoated samples than for the Li–PIL coated samples. Therefore, it can be concluded that the Li–PIL coatings on NMC82 reduce the amount of decomposition of the Li_6_PS_5_Cl solid-electrolyte at the NMC82 interface. Furthermore, the XPS spectra of pre-cycling and post-cycling PIL coated samples show identical trends as the Li–PIL coated samples (Fig. S19†), suggesting that the interfacial stability imparted by (Li)PIL coatings to NMC82 in sulfide SSBs might be independent of the Li salt concentration.

In addition, the F 1s and N 1s XPS spectra for the composites after cycling (Fig. S20a and b†) reveal minimal changes to the Li–PIL coating environment with cycling (compared to Fig. 2d and e). To further verify this, we performed ^19^F and ^13^C NMR on the cathode composites of coated NMC82 before and after cycling (Fig. S20c and d†). For the ^19^F NMR, the environment corresponding to TFSI^−^ is still present in the post-cycled spectrum with no additional environments, which is consistent with the F 1s XPS results. With ^13^C NMR, a similar ratio of PDDA^+^/TFSI^−^ peaks is observed before and after cycling, which aligns well with the N 1s XPS results.

It has to be noted that the post-cycling samples have a lower degree of paramagnetism at a partially delithiated state of NMC82 (3.62 V *vs.* Li^+^/Li) as compared to the pristine samples. This results in lower PDDA^+^ peak broadening in the case of ^13^C NMR and a narrower sideband manifold width in the case of ^19^F NMR. Furthermore, the ^13^C NMR spectra of the cathode composites were acquired with a proton decoupling power of 30 W and a spinning speed of 35 kHz. Therefore, the ratio of PDDA^+^/TFSI^−^ peaks is not comparable to that of the coated powder (Fig. 2g, 60 W proton decoupling power, 20 kHz spinning speed). Overall, our findings indicate that the applied Li–PIL coating is (electro)chemically stable with long-term cycling.

### 3.5 Effect on mechanical and structural stability

**Fig. 6:**
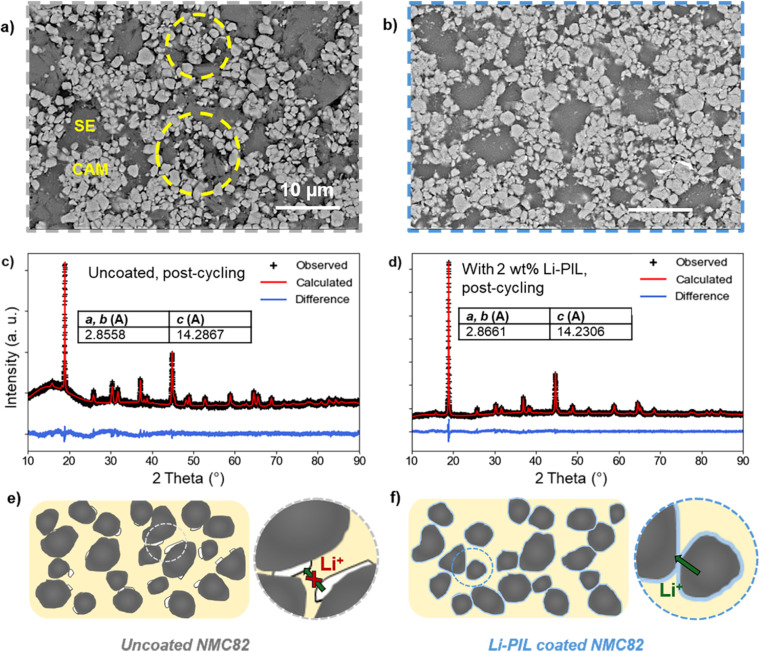
Top: SEM images of the NMC82/Li_6_PS_5_Cl composites with; (a) NMC82 after cycling and (b) NMC82 with Li–PIL after cycling. All images were taken with BED, and a 15 kV acceleration voltage, with a magnification of 2500×. Yellow circles indicate the contact loss after cycling for uncoated NMC82. Middle: Post-cycling XRD patterns of (c) cathode composite with uncoated NMC82 (d) cathode composite with 2 wt% Li–PIL coated NMC82. Bottom: (e and f) Provide schematic overview of the evolution of mechanical contact at the interfaces for LPSC/NMC82 composites without and with the Li–PIL coating respectively.

A key advantage of polymeric surface coatings for cathodes in SSBs is their ability to buffer volume changes during cycling.^18,36^ This is also reflected in the post-cycling SEM images of the uncoated and coated cathode composites (Fig. 6 and S21†).

In the case of uncoated NMC82, severe contact loss with several voids in the cathode composite is observed after cycling (Fig. 6a). The yellow circles indicate the areas with significant contact loss. In contrast, for cathode composites with Li–PIL (Fig. 6b) and PIL coatings (Fig. S21b†), the contact between the CAM and SE particles is still maintained after cycling, likely due to the volume buffering capacity of the polymer coating during the lithiation and delithiation of the NMC particles. The limited number of visible voids of the post-cycled samples compared to the pre-cycling uncoated NMC82 cathode composite (Fig. S21a†) indicates that initial particle contact could have been improved by the polymeric coating as well.

As indicated by the compression tests carried out on Li–PIL film (Fig. S22,† 600 μm film subjected to a compression of up to 100 μm at 2 μm s^−1^), under uniaxial compression, the Li–PIL behaves as a viscoelastic solid. About ∼37% of the imposed strain is recovered elastically, while the hysteresis between the compression and recovery curves indicates that the polymer also undergoes some viscous flow/reconfiguration. Together, this indicates that the Li–PIL could be counteracting the differential stress experienced during lithiation/delithiation through a combination of elastic recovery and viscous dissipation, although elucidating its precise effect when used as a nanocoating on metal oxides like NMC82 would require a more detailed study.

As Ni-rich layered oxides can be highly prone to volume change-induced structural instabilities upon continuous cycling, we also performed XRD on coated and uncoated NMC82 cathode composites post-cycling to observe differences, if any, in the crystal structure retention (Fig. 6c and d). The Pawley refinement results show a slightly greater (003) peak shift for the uncoated NMC82, as well as slightly increased peak broadening (Fig. S23†), associated with the presence of microstrain on the uncoated NMC.^62^ On comparing the lattice parameters before and after cycling (Fig. S14† and 6c, d), it is observed that the lattice parameters of the Li–PIL coated NMC82 show a lower degree of change with cycling compared to the uncoated NMC82 (*c* parameter: 14.2867 Å for uncoated *vs.* 14.2306 Å for Li–PIL coated). While NMC particles are known to experience microstrain only at very high states of charge (delithiation),^63^ it is plausible that the volume buffering offered by the polymeric coatings (as shown with SEM) alleviates the heterogeneities in the degree of (de)lithiation in the cathode composite otherwise caused due to contact loss, thereby reducing the overall extent of microstrain on the NMC particles present in the cathode composite.

To summarize, while uncoated NMC82 suffers from cycling-induced contact loss, resulting in poor ionic percolation through the cathode composite and capacity loss, the presence of Li–PIL coating on NMC82 improves contact retention between NMC82 and SE particles with cycling, and given the ion-conductive nature of the coating, this further helps in the retention of the ionic percolation pathways through the cathode composite (Fig. 6e and f).

## 4 Conclusions

In this work, we investigate the incorporation of Li salt into polymerized ionic liquid surface coatings as a strategy for simultaneously addressing contact losses, electrochemical decomposition and Li^+^ transport bottlenecks over the cathode–electrolyte interface in sulfide-based solid-state batteries with Ni-rich layered oxide cathodes. For the polymerized ionic liquid PDDATFSI, we show that the inclusion of LiTFSI in a 1 : 1 (mol%) ratio results in a good balance of Li^+^ diffusivity and transference, as shown by PFG-NMR and electrochemical results. This translates to minimal Li^+^ concentration and potential drops across the polymer at practical current densities, as shown by our analytical modelling results. The chemical compatibility of Li–PIL with the sulfide electrolyte Li_6_PS_5_Cl also results in facile Li^+^ transport across the Li_6_PS_5_Cl/Li–PIL interface, as demonstrated by ^7^Li 2D exchange NMR.

With 2 wt% Li–PIL (of the cathode active mass), thin nanocoatings of Li–PIL are obtained on Ni-rich NMC82, as confirmed by TEM, XPS, and ssNMR measurements. On testing in SSBs, the Li–PIL coating on NMC82 improves both the rate capability (190 mA h g^−1^*vs.* 163 mA h g^−1^ for uncoated at 0.1C, 92.7 mA h g^−1^*vs.* 76.6 mA h g^−1^ for uncoated at 1C) and the capacity retention (95% *vs.* 84% after 100 cycles at 0.2C), while the PIL coating without LiTFSI improves only the capacity retention (94% after 100 cycles) with a reduction in the initial discharge capacity. An exceptional capacity retention of 82.7% is observed for the Li–PIL coated NMC82 after 500 cycles at 0.2C under ambient conditions (20 °C).

EIS measurements show minimal resistance evolution across the cathode electrolyte interface for the Li–PIL-coated sample, compared to a severe resistance increase for uncoated NMC82. Post-cycling XPS results show that the Li–PIL coating greatly reduces electrolyte oxidation at the NMC82/Li_6_PS_5_Cl interface. Furthermore, the presence of Li–PIL coatings also significantly improves CAM/SE particle contact retention with cycling, as established by the SEM images. In addition, the XRD results also indicate that the Li–PIL coating reduces the overall degree of structural deformation in the cathode composite, likely due to the improvements in contact retention and the preservation of ionic percolation pathways through the cathode composite. Our results establish Li salt incorporation into polymerized ionic liquid surface coatings as a highly effective strategy for overcoming both chemomechanical challenges and ion transport bottlenecks in sulfide-based solid-state batteries with Ni-rich layered oxide cathodes, particularly under ambient temperature conditions.

## Author contributions

Pranav Karanth: conceptualization, investigation, methodology, validation, writing – original draft. Jelle H. Prins: investigation, methodology, validation, writing – original draft. Ajay Gautam: methodology, validation, writing – review & editing. Zhu Cheng: methodology, writing – review & editing. Jef Canals-Riclot: validation, writing – review & editing. Swapna Ganapathy: resources, supervision, writing – review & editing. Pierfrancesco Ombrini: formal analysis, methodology, writing – original draft. Alix Ladam: resources, writing – review & editing. Sebastien Fantini: resources, writing – review & editing. Marnix Wagemaker: resources, supervision, writing – review & editing. Fokko M. Mulder: conceptualization, project administration, validation, supervision, writing – review & editing.

## Conflicts of interest

The authors declare no conflict of interest.

## Supplementary Material

TA-013-D5TA01827G-s001

## Data Availability

The data supporting this article have been included as part of the ESI.† Additional raw data can be obtained from the corresponding authors upon request.

## References

[cit1] Janek J., Zeier W. G. (2016). Nat. Energy.

[cit2] Han Y., Jung S. H., Kwak H., Jun S., Kwak H. H., Lee J. H., Hong S.-T., Jung Y. S. (2021). Adv. Energy Mater..

[cit3] Chen S., Xie D., Liu G., Mwizerwa J. P., Zhang Q., Zhao Y., Xu X., Yao X. (2018). Energy Storage Mater..

[cit4] Kerman K., Luntz A., Viswanathan V., Chiang Y.-M., Chen Z. (2017). J. Electrochem. Soc..

[cit5] Pasta M., Armstrong D., Brown Z. L., Bu J., Castell M. R., Chen P., Cocks A., Corr S. A., Cussen E. J., Darnbrough E., Deshpande V., Doerrer C., Dyer M. S., El-Shinawi H., Fleck N., Grant P., Gregory G. L., Grovenor C., Hardwick L. J., Irvine J. T. S., Lee H. J., Li G., Liberti E., McClelland I., Monroe C., Nellist P. D., Shearing P. R., Shoko E., Song W., Jolly D. S., Thomas C. I., Turrell S. J., Vestli M., Williams C. K., Zhou Y., Bruce P. G. (2020). JPhys Energy.

[cit6] Koerver R., Zhang W., de Biasi L., Schweidler S., Kondrakov A. O., Kolling S., Brezesinski T., Hartmann P., Zeier W. G., Janek J. (2018). Energy Environ. Sci..

[cit7] Janek J., Zeier W. G. (2023). Nat. Energy.

[cit8] Minnmann P., Strauss F., Bielefeld A., Ruess R., Adelhelm P., Burkhardt S., Dreyer S. L., Trevisanello E., Ehrenberg H., Brezesinski T., Richter F. H., Janek J. (2022). Adv. Energy Mater..

[cit9] Wang C., Yu R., Hwang S., Liang J., Li X., Zhao C., Sun Y., Wang J., Holmes N., Li R., Huang H., Zhao S., Zhang L., Lu S., Su D., Sun X. (2020). Energy Storage Mater..

[cit10] Gregory G. L., Gao H., Liu B., Gao X., Rees G. J., Pasta M., Bruce P. G., Williams C. K. (2022). J. Am. Chem. Soc..

[cit11] Koerver R., Aygün I., Leichtweiß T., Dietrich C., Zhang W., Binder J. O., Hartmann P., Zeier W. G., Janek J. (2017). Chem. Mater..

[cit12] Tan D. H. S., Wu E. A., Nguyen H., Chen Z., Marple M. A. T., Doux J.-M., Wang X., Yang H., Banerjee A., Meng Y. S. (2019). ACS Energy Lett..

[cit13] Walther F., Koerver R., Fuchs T., Ohno S., Sann J., Rohnke M., Zeier W. G., Janek J. (2019). Chem. Mater..

[cit14] Walther F., Strauss F., Wu X., Mogwitz B., Hertle J., Sann J., Rohnke M., Brezesinski T., Janek J. (2021). Chem. Mater..

[cit15] Kitsche D., Tang Y., Hemmelmann H., Walther F., Bianchini M., Kondrakov A., Janek J., Brezesinski T. (2023). Small Sci..

[cit16] Ma Y., Zhang R., Tang Y., Ma Y., Teo J. H., Diemant T., Goonetilleke D., Janek J., Bianchini M., Kondrakov A., Brezesinski T. (2022). ACS Nano.

[cit17] Kitsche D., Tang Y., Ma Y., Goonetilleke D., Sann J., Walther F., Bianchini M., Janek J., Brezesinski T. (2021). ACS Appl. Energy Mater..

[cit18] Amin R., Nisar U., Rahman M. M., Dixit M., Abouimrane A., Belharouak I. (2024). J. Mater. Chem. A.

[cit19] Sen S., Trevisanello E., Niemöller E., Shi B.-X., Simon F. J., Richter F. H. (2021). J. Mater. Chem. A.

[cit20] Xu G.-L., Liu Q., Lau K. K. S., Liu Y., Liu X., Gao H., Zhou X., Zhuang M., Ren Y., Li J., Shao M., Ouyang M., Pan F., Chen Z., Amine K., Chen G. (2019). Nat. Energy.

[cit21] Deng S., Sun Y., Li X., Ren Z., Liang J., Doyle-Davis K., Liang J., Li W., Norouzi Banis M., Sun Q., Li R., Hu Y., Huang H., Zhang L., Lu S., Luo J., Sun X. (2020). ACS Energy Lett..

[cit22] Gan Q., Qin N., Zhu Y., Huang Z., Zhang F., Gu S., Xie J., Zhang K., Lu L., Lu Z. (2019). ACS Appl. Mater. Interfaces.

[cit23] Lin C., Liu Y., Su H., Zhong Y., Wang X., Gu C., Tu J. (2024). Adv. Funct. Mater..

[cit24] Shi B.-X., Weber F., Yusim Y., Demuth T., Vettori K., Münchinger A., Titvinidze G., Volz K., Henss A., Berger R., Richter F. H. (2025). J. Mater. Chem. A.

[cit25] Sun B., Kazzi M. E., Müller E., Berg E. J. (2018). J. Mater. Chem. A.

[cit26] Eshetu G. G., Mecerreyes D., Forsyth M., Zhang H., Armand M. (2019). Mol. Syst. Des. Eng..

[cit27] Appetecchi G. B., Kim G.-T., Montanino M., Carewska M., Marcilla R., Mecerreyes D., De Meatza I. (2010). J. Power Sources.

[cit28] Wang X., Zhu H., Girard G. M. A., Yunis R., MacFarlane D. R., Mecerreyes D., Bhattacharyya A. J., Howlett P. C., Forsyth M. (2017). J. Mater. Chem. A.

[cit29] Wang X., Chen F., Girard G. M. A., Zhu H., MacFarlane D. R., Mecerreyes D., Armand M., Howlett P. C., Forsyth M. (2019). Joule.

[cit30] Song X., Wang C., Chen J., Xin S., Yuan D., Wang Y., Dong K., Yang L., Wang G., Zhang H., Zhang S. (2022). Adv. Funct. Mater..

[cit31] Vauthier S., Alvarez-Tirado M., Guzmán-González G., Tomé L. C., Cotte S., Castro L., Guéguen A., Mecerreyes D., Casado N. (2023). Mater. Today Chem..

[cit32] Fu C., Homann G., Grissa R., Rentsch D., Zhao W., Gouveia T., Falgayrat A., Lin R., Fantini S., Battaglia C. (2022). Adv. Energy Mater..

[cit33] Homann G., Wang Q., Liu S., Devincenti A., Karanth P., Weijers M., Mulder F. M., Piesins M., Gouveia T., Ladam A., Fantini S., Battaglia C. (2024). ACS Appl. Energy Mater..

[cit34] Li L., Pascal T. A., Connell J. G., Fan F. Y., Meckler S. M., Ma L., Chiang Y.-M., Prendergast D., Helms B. A. (2017). Nat. Commun..

[cit35] Fan X., Wang Y., Zeng M., He H., Huang J., Feng Z., Li J., Liang Z., Zhou T. (2022). J. Alloys Compd..

[cit36] Shi B.-X., Yusim Y., Sen S., Demuth T., Ruess R., Volz K., Henss A., Richter F. H. (2023). Adv. Energy Mater..

[cit37] Santhosha A. L., Medenbach L., Buchheim J. R., Adelhelm P. (2019). Batteries Supercaps.

[cit38] Bruce P. G., Evans J., Vincent C. A. (1988). Solid State Ionics.

[cit39] De Vos N., Maton C., Stevens C. V. (2014). ChemElectroChem.

[cit40] Zhao Q., Bennington P., Nealey P. F., Patel S. N., Evans C. M. (2021). Macromolecules.

[cit41] Cheng J., Fong K. D., Persson K. A. (2022). J. Mater. Chem. A.

[cit42] Popov I., Biernacka K., Zhu H., Nti F., Porcarelli L., Wang X., Khamzin A., Gainaru C., Forsyth M., Sokolov A. P. (2020). J. Phys. Chem. C.

[cit43] Stacy E. W., Gainaru C. P., Gobet M., Wojnarowska Z., Bocharova V., Greenbaum S. G., Sokolov A. P. (2018). Macromolecules.

[cit44] Haverkort W. , Electrolysers, Fuel Cells and Batteries: Analytical Modelling, TU Delft OPEN Textbooks, 2024

[cit45] Vargas-Barbosa N. M., Roling B. (2020). ChemElectroChem.

[cit46] Liu M., Zhang S., van Eck E. R. H., Wang C., Ganapathy S., Wagemaker M. (2022). Nat. Nanotechnol..

[cit47] Simon F. J., Hanauer M., Henss A., Richter F. H., Janek J. (2019). ACS Appl. Mater. Interfaces.

[cit48] Wagemaker M., Kentgens A. P. M., Mulder F. M. (2002). Nature.

[cit49] Yu C., Ganapathy S., van Eck E. R. H., Wang H., Basak S., Li Z., Wagemaker M. (2017). Nat. Commun..

[cit50] Liu M., Wang C., Zhao C., van der Maas E., Lin K., Arszelewska V. A., Li B., Ganapathy S., Wagemaker M. (2021). Nat. Commun..

[cit51] Yu W., Yu Z., Cui Y., Bao Z. (2022). ACS Energy Lett..

[cit52] Weber I., Kim J., Buchner F., Schnaidt J., Behm R. J. (2020). ChemSusChem.

[cit53] Wickham J. R., Mason R. N., Rice C. V. (2007). Solid State Nucl. Magn. Reson..

[cit54] Zhou Y.-N., Ma J., Hu E., Yu X., Gu L., Nam K.-W., Chen L., Wang Z., Yang X.-Q. (2014). Nat. Commun..

[cit55] Lu Y., Zhao C.-Z., Huang J.-Q., Zhang Q. (2022). Joule.

[cit56] Yu C.-Y., Choi J., Dunham J., Ghahremani R., Liu K., Lindemann P., Garver Z., Barchiesi D., Farahati R., Kim J.-H. (2024). J. Power Sources.

[cit57] Zhao W., Zhang R., Ren F., Karger L., Dreyer S. L., Lin J., Ma Y., Cheng Y., Pal A. S., Velazquez-Rizo M., Ahmadian A., Zhang Z., Müller P., Janek J., Yang Y., Kondrakov A., Brezesinski T. (2025). ACS Nano.

[cit58] Puls S., Nazmutdinova E., Kalyk F., Woolley H. M., Thomsen J. F., Cheng Z., Fauchier-Magnan A., Gautam A., Gockeln M., Ham S.-Y., Hasan M. T., Jeong M.-G., Hiraoka D., Kim J. S., Kutsch T., Lelotte B., Minnmann P., Miß V., Motohashi K., Nelson D.
L., Ooms F., Piccolo F., Plank C., Rosner M., Sandoval S. E., Schlautmann E., Schuster R., Spencer-Jolly D., Sun Y., Vishnugopi B. S., Zhang R., Zheng H., Adelhelm P., Brezesinski T., Bruce P. G., Danzer M., El Kazzi M., Gasteiger H., Hatzell K. B., Hayashi A., Hippauf F., Janek J., Jung Y. S., McDowell M. T., Meng Y. S., Mukherjee P. P., Ohno S., Roling B., Sakuda A., Schwenzel J., Sun X., Villevieille C., Wagemaker M., Zeier W. G., Vargas-Barbosa N. M. (2024). Nat. Energy.

[cit59] Wu X., Mirolo M., Vaz C. A. F., Novák P., El Kazzi M. (2021). ACS Appl. Mater. Interfaces.

[cit60] Walther F., Randau S., Schneider Y., Sann J., Rohnke M., Richter F. H., Zeier W. G., Janek J. (2020). Chem. Mater..

[cit61] Wang S., Tang M., Zhang Q., Li B., Ohno S., Walther F., Pan R., Xu X., Xin C., Zhang W., Li L., Shen Y., Richter F. H., Janek J., Nan C.-W. (2021). Adv. Energy Mater..

[cit62] Chung H., Li Y., Zhang M., Grenier A., Mejia C., Cheng D., Sayahpour B., Song C., Shen M. H., Huang R., Wu E. A., Chapman K. W., Kim S. J., Meng Y. S. (2022). Chem. Mater..

[cit63] Ryu H.-H., Park N.-Y., Noh T.-C., Kang G.-C., Maglia F., Kim S.-J., Yoon C. S., Sun Y.-K. (2021). ACS Energy Lett..

