# Supplementary material for: Multifunctional ion-conductive polymer coatings for high-performance sulfide solid-state batteries with Ni-rich cathodes
Source: J Mater Chem A Mater. 2025 May 13;13(24):18518–31. doi: 10.1039/d5ta01827g (PMC12086626; doi:10.1039/d5ta01827g)
Supplement: TA-013-D5TA01827G-s001 [file TA-013-D5TA01827G-s001.pdf]

Supplementary Information (SI) for Journal of Materials Chemistry A.
This journal is © The Royal Society of Chemistry 2025

Supporting Information

Multifunctional ion-conductive polymer coatings for high-performance sulfide solid-state batteries with Ni-rich cathodes

Pranav Karanth[a], Jelle H. Prins[a], Ajay Gautam[b], Zhu Cheng[b], Jef Canals-Riclot[b], Swapna Ganapathy[b], Pierfrancesco Ombrini[b], Alix Ladam[c], Sebastien Fantini[c], Marnix Wagemaker[b] and Fokko M. Mulder[a,*]

[a] Department of Chemical Engineering, Delft University of Technology, The Netherlands.

*Email: F.m.mulder@tudelft.nl

[b] Department of Radiation Science and Technology, Delft University of Technology, The Netherlands.

[c] Solvionic, 11 Chemin des Silos, Toulouse 31100, France.

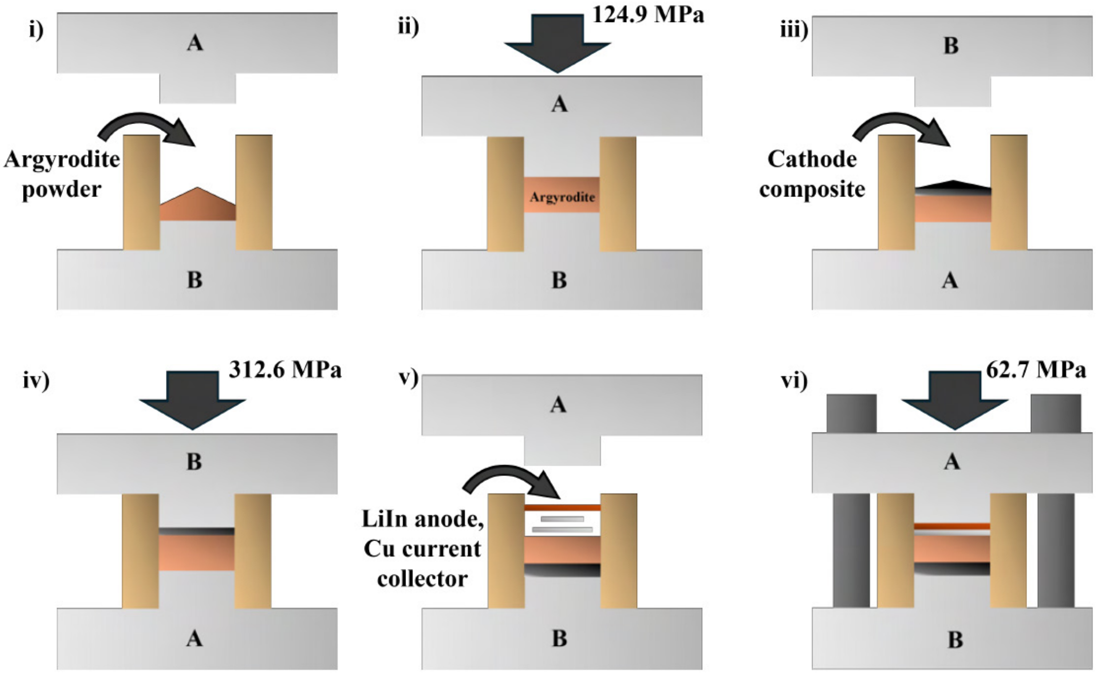

Figure S1: Schematic representation of the solid-state battery assembly procedure.

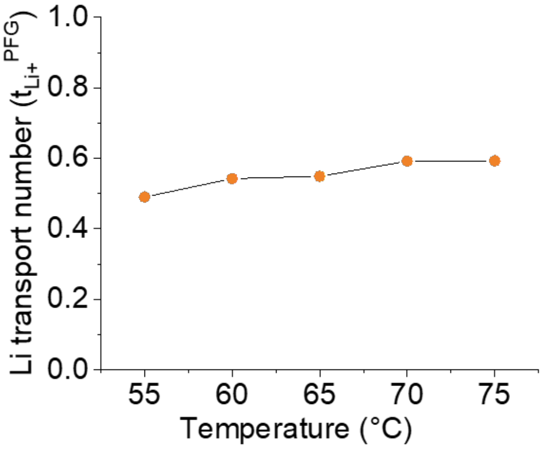

Figure S2: Li* transport number of 1:1 Li-PIL (obtained from PFG NMR) as a function of temperature

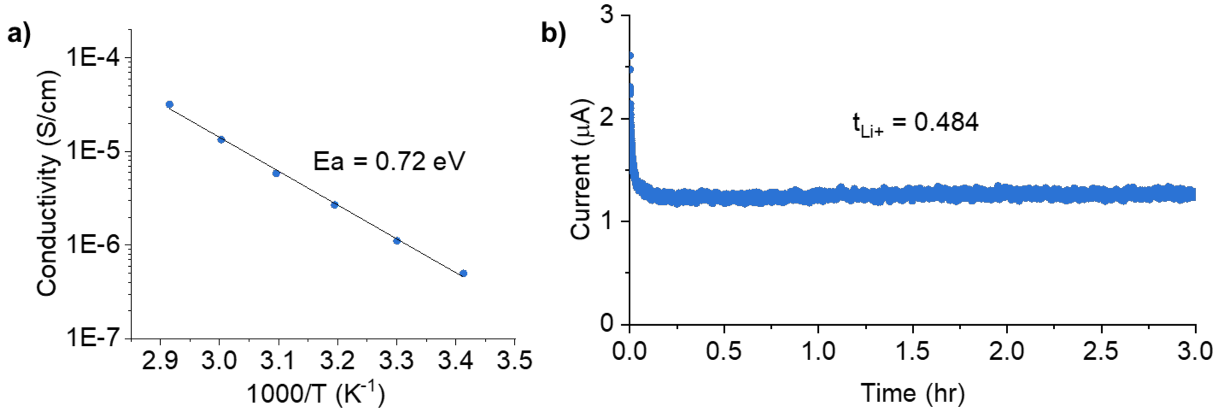

Figure S3: a) Ionic conductivity of 1:1 Li-PIL as a function of temperature and b) Chronoamperometry profile under a DC polarization of 5 mV for the determination of Bruce-Vincent Li^+ transference number at 60 °C

Supplementary Note 1: Calculation of Inverse Haven Ratios

In polymer electrolytes, the PFG NMR diffusivities (Li* and F* in this case) represent self-diffusion coefficients under equilibrium (i.e., zero applied electric field) conditions, while the electrochemically measured ionic conductivity reflects the total ion conduction under an applied field, including ion correlations that can enhance or suppress net ionic flow. These ion-ion correlations are captured by the Haven ratio (or alternatively, the inverse Haven ratio, i.e. the ratio of electrochemically measured conductivity to the self-diffusivity-based conductivity).[1] Understanding both aspects is essential for understanding and optimizing Li^+ transport, since high self-diffusion rates do not always result in high bulk conductivity if strong ion-ion correlations impede net ion flow.

The scheme for obtaining the Inverse Haven ratio (H^{-1}) is as follows, based on PFG self-diffusivity and electrochemical conductivity values at 333 K:

- $D_{Li^*} = 6.18\times10^{-13}\ m^2/s$
- $D_{F^*} = 4.43\times10^{-13}\ m^2/s$
- Electrochemically measured conductivity, σ_{ele}=1.34×10^{-5} S/cm
- Molarity (based on density measurements and chosen mole ratio), c ≈ 2 M = 2000 mol/m^3
- Faraday's constant, F = 96485 C/mol
- Gas constant, R = 8.314 J/mol. K

For a fully dissociated salt, the 'uncorrelated' Nernst–Einstein conductivity is given by:

$$\sigma_{NE} = \frac{c\,F^2\,(D_{Li^*} + D_{F^*})}{RT} \approx 7.12\times10^{-5}\ \text{S/cm}$$

The Inverse Haven ratio is then obtained as:

$$H^{-1} = \frac{\sigma_{ele}}{\sigma_{NE}} = \frac{1.34\times10^{-5}}{7.12\times10^{-5}} \approx 0.19.$$

Similarly, at 343 K , σ_{ele}=3.19×10^{-5} S/cm, the obtained $\sigma_{NE} \approx 1.39\times10^{-4}$ S/cm, and $H^{-1} = 0.23$.

Supplementary Note 2: Analytical solution of concentration and potential drop profiles in the Li-PIL coating

To evaluate the concentration and potential drop profiles in the Li-PIL coating, the Li-PIL coating is assumed to be an ideal binary electrolyte layer of thickness δ, diffusivity D_+ and concentration c_0. The steady-state concentration profile in the interface c_e can be calculated as a function of distance x (from the solid electrolyte interface), based on the Nernst-Planck transport equation,[2] as:

$$c_e(x) = c_0 - \frac{j}{2\,F\,D_+}\left(x - \frac{\delta}{2}\right)$$

Where F is the Faraday constant and j is the specific current density, obtained by assuming a uniform current distribution across all the particle surfaces.

The potential drop $\Delta\phi$, defined as the difference between the potential at the solid electrolyte-coating interface and the coating-NMC82 interface, can also be analytically calculated as:

$$\Delta\phi(x) = \frac{RT}{F}\log\left(1 - \frac{\frac{2}{\delta}x}{1 + \frac{2\,c_0\,F\,D_+}{j\,\delta}}\right)$$

It is to be noted however, that the assumption of ideal binary electrolyte is an approximation in the case of polymer electrolytes with high salt concentrations, as these systems typically also include cation-anion correlated motions, resulting in deviations from this ideal behavior.[1,3] Furthermore, ion diffusivity in thin film polymers is also known to drop due to confinement effects.[4] Therefore, while the extrapolation of diffusivities from PFG NMR (Figure 6a) results in Li* diffusivity of around 4 x 10^{-14} m^2/s at 20 °C, we assume a conservative lower bound of 10^{-15} m^2/s for D_+.

An overview of the values of parameters used for Figures 1b and 1c is shown below.

Table S1: Overview of parameters and values used for steady-state concentration and potential drop calculations

Parameter	Value, units
Cationic diffusivity, D_+	10^{-15} m^2/s
Anionic diffusivity, D_-	4 x10^{-15} m^2/s
Li-PIL thickness δ	100 nm
Li-PIL density (measured for ~300 µm film)	~ 1.4 g/cm^3
Li+ initial concentration in Li-PIL, c_0	2 M (or mol/dm^3)
Temperature, T	293 K
Total cycled capacity	2 mAh/cm^2
Specific surface area of NMC82	0.3 m^2/g

The concentration polarization occurs at the beginning of the discharge process since its characteristic time is $\tau = \delta^2/D_{amb}$, where $D_{amb} = 2\frac{D_+D_-}{D_++D_-}$. For the case of D_+ = 10^{-15} m^2/s and D_- = 4 x 10^{-15} m^2/s , the ambipolar diffusivity D_{amb} = 1.6 x 10^{-15} m^2/s. The relaxation time for a layer of thickness $\delta =$ 100 nm is then τ = 6.25 s. For $\delta =$ 10 nm, τ = 0.06 s. This means the Li^+ concentration in the coating quickly equilibrates at the beginning of the lithiation process, reaching the steady-state solution provided above.

Furthermore, the influence of D_+ on concentration polarization and potential drops at different coating thicknesses is evaluated (Figure S4 a,b). Here, it can be observed that for a thickness of 10 nm, the target thickness of this work, these effects become significant only for D_+ values below 10^{-16} m^2/s (with a current density of 2 mA/cm^2).

When it comes to optimizing the coating thickness, while a thicker coating could offer increased surface protection, the potential/concentration drop across the same would negatively influence the cell overpotential/rate capability. The influence of coating thickness on concentration polarization and potential drops at different current densities (for a total capacity of 2 mAh/cm^2) is also evaluated (Figure S4 c, d). Here, it can be observed that the limiting thicknesses vary depending on the targeted current densities, with a thickness of about 200 nm being the limit for the highest current density probed (10 mA/cm^2).

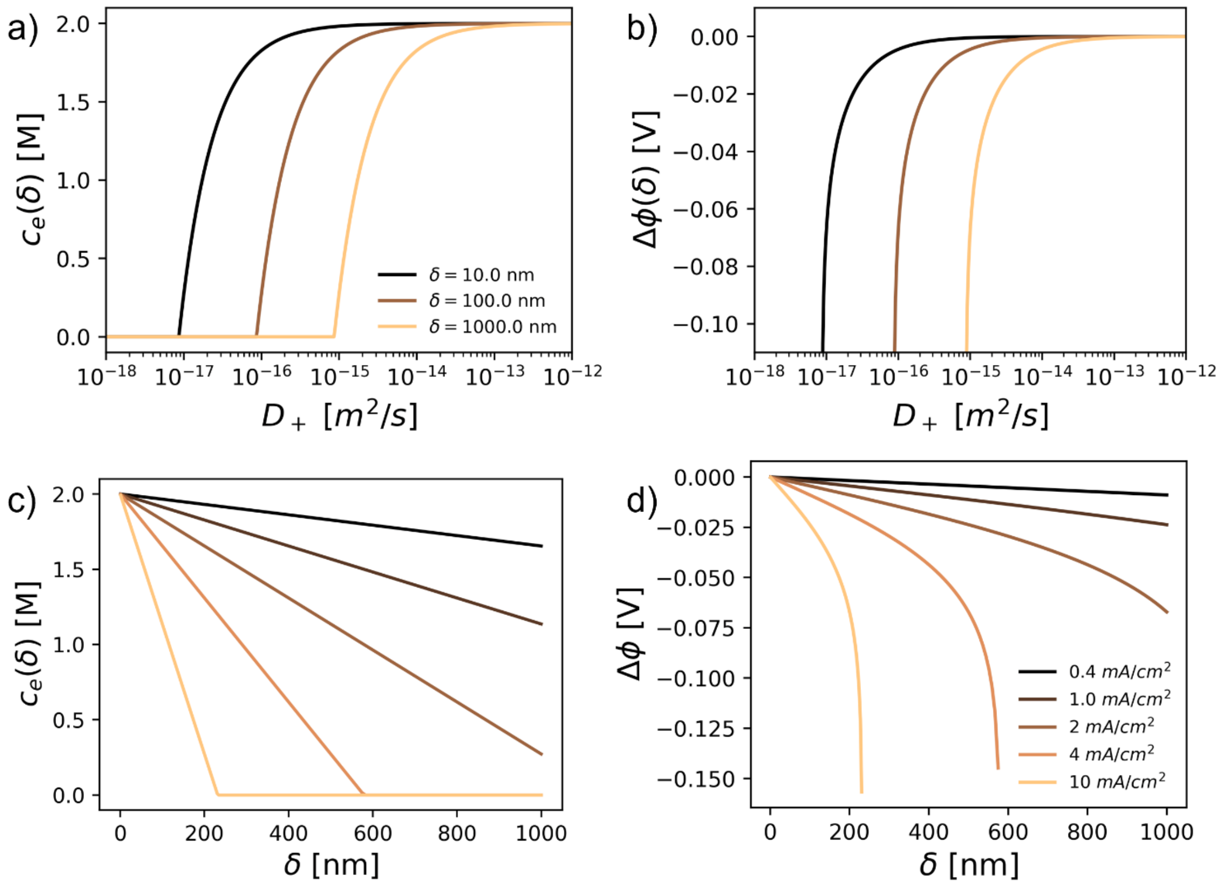

Figure S4: a) Steady-state electrolyte concentration at the cathode/Li-PIL interface and b) potential drop across the polymer coating as a function of Li^+ diffusivity at different Li-PIL coating thicknesses. c) Steady-state electrolyte concentration at the cathode/Li-PIL interface and d) potential drop across the polymer coating as a function of Li-PIL coating thickness at different current densities.

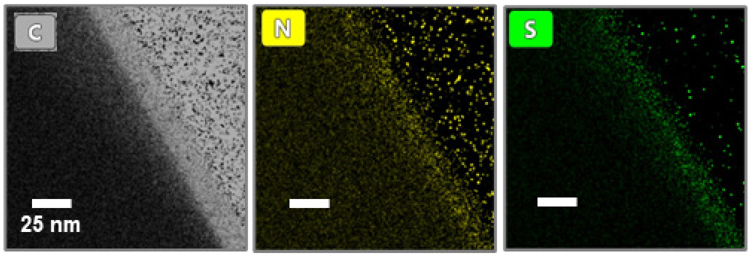

Figure S5: STEM-EDX elemental at% mapping of C, N and S, for Li-PIL coated on NMC82 (corresponding to the HAADF-STEM image shown in Figure 2b)

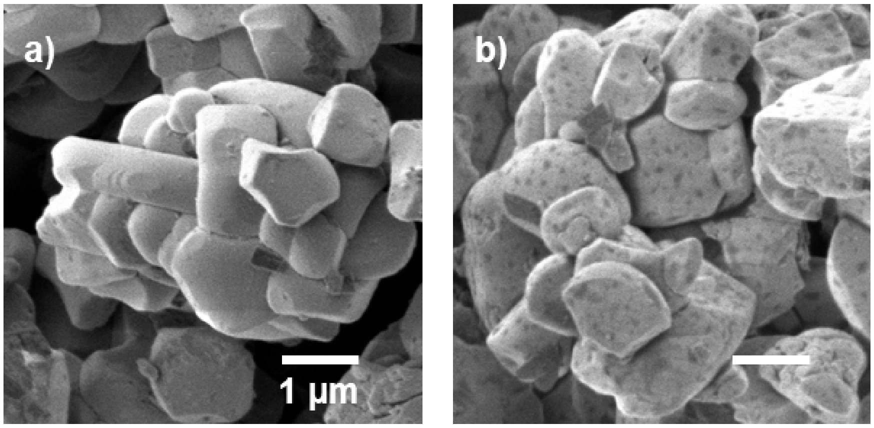

Figure S6: a) SEM image of NMC82 and b) SEM image of NMC82 with 2 wt% Li-PIL coating, a and b were taken with SED mode, and 1kV acceleration voltage.

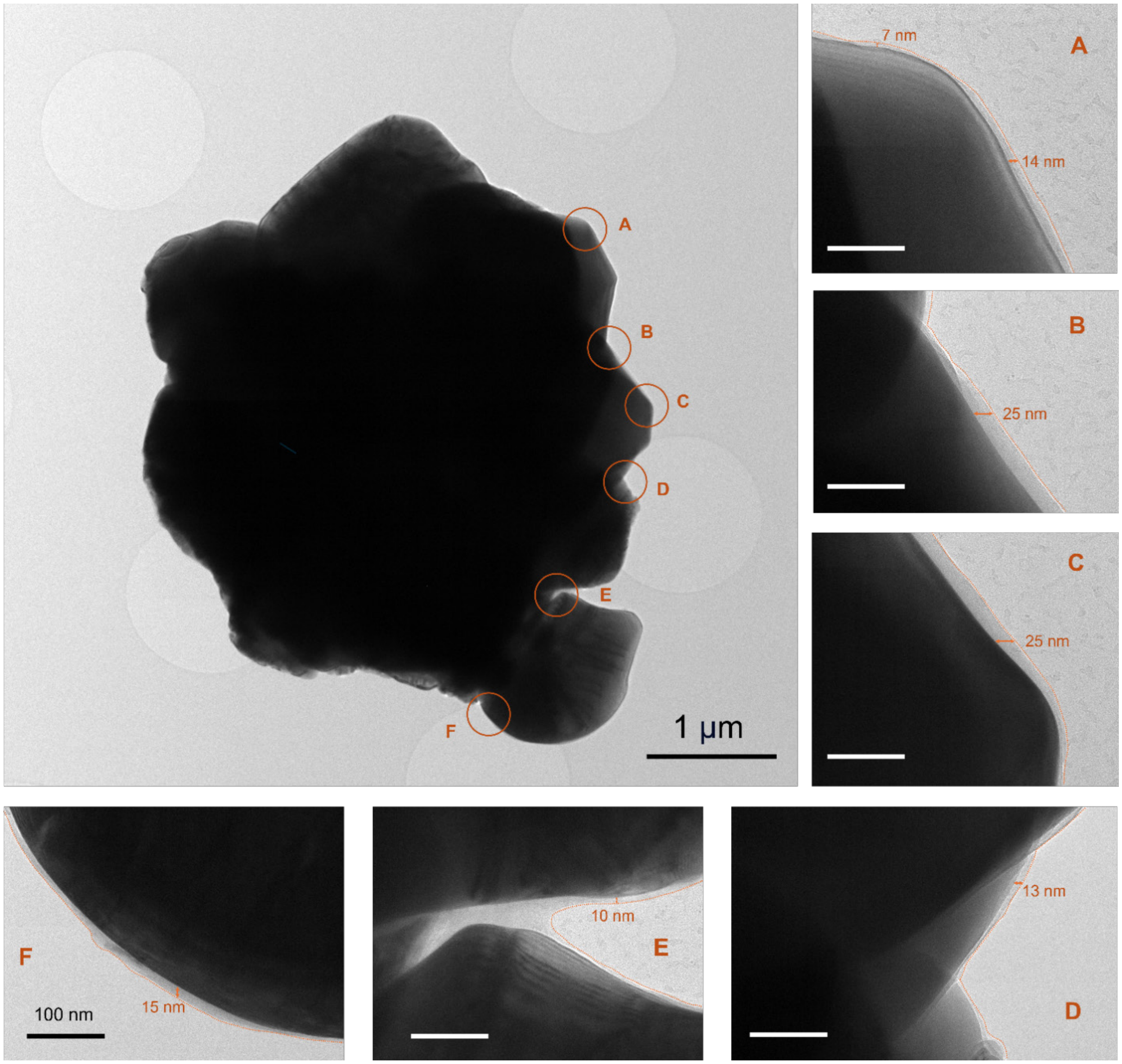

Figure S7: TEM image of an NMC82 agglomerate coated with 2 wt% Li-PIL. Zoomed images for regions A-F on the agglomerate show the Li-PIL coating with a thickness range of 5-25 nm, with higher thickness values between NMC82 grains (Region E). The scale bar is 1 μm for the main figure and 100 nm for regions A-F.

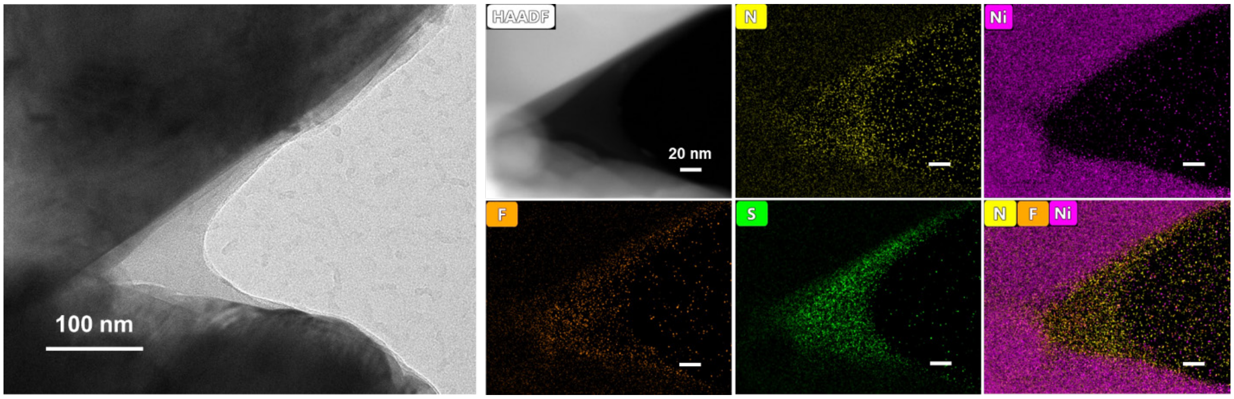

Figure S8: TEM image of Li-PIL between NMC82 grains (left) and HAADF, and STEM-EDX elemental at% mapping of Ni, Mn, F, and S, for Li-PIL between NMC82 grains (right). The scale bar for the EDX maps is 20 nm

a)

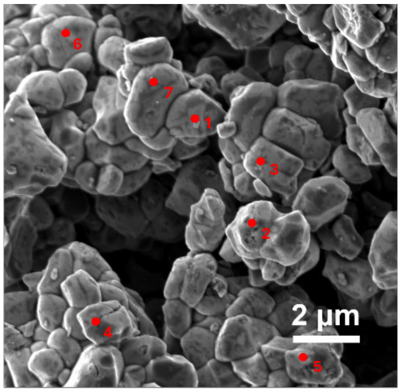

Site	C – K (wt %)	N – K (wt %)	O - K (wt %)	F – K (wt %)	S – K (wt %)	Mn – L (wt %)	Co – L (wt %)	Ni – L (wt %)
1	0.56	0.28	30.52	0.67	0.29	5.63	9.7	52.35
2	0.71	0.45	28.78	0.92	0.24	6.6	9.86	52.44
3	0.81	0.38	28.55	0.9	0.15	5.73	10.35	52.13
4	1.9	0.67	28.67	1.45	0.49	4.14	10.25	52.43
5	0.47	0.43	28.67	0.8	0.07	4.6	11.07	53.89
6	0.42	0.32	29.49	0.92	0.24	5.56	9.49	53.56
7	0.64	0.42	29.03	0.85	0.08	6.54	9.64	52.81

b)

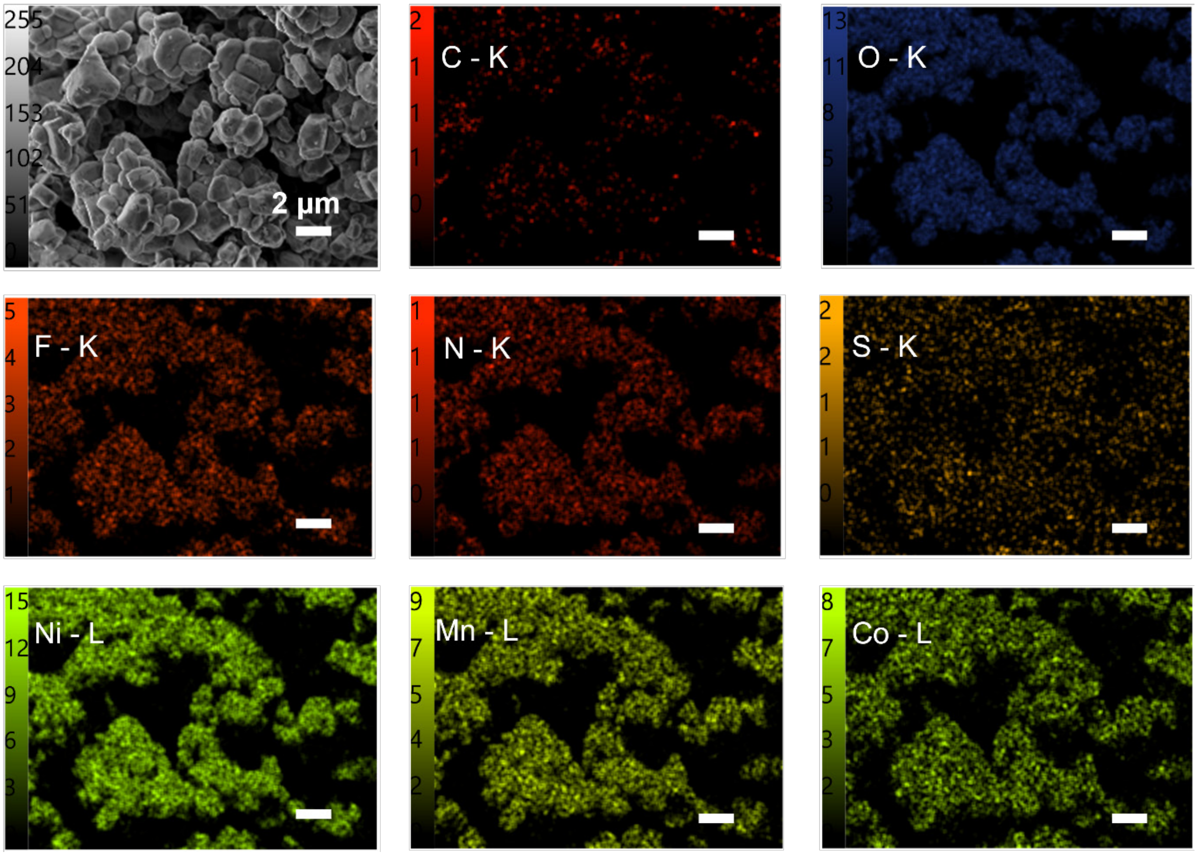

Figure S9: a) EDS point analysis for NMC82 coated with Li-PIL. The sites 1-7 are indicated on the left image, and the corresponding elemental wt% values are provided on the right. b) EDS elemental mapping for NMC82 coated with Li-PIL. Both a) and b) were acquired at an electron acceleration voltage of 5 kV.

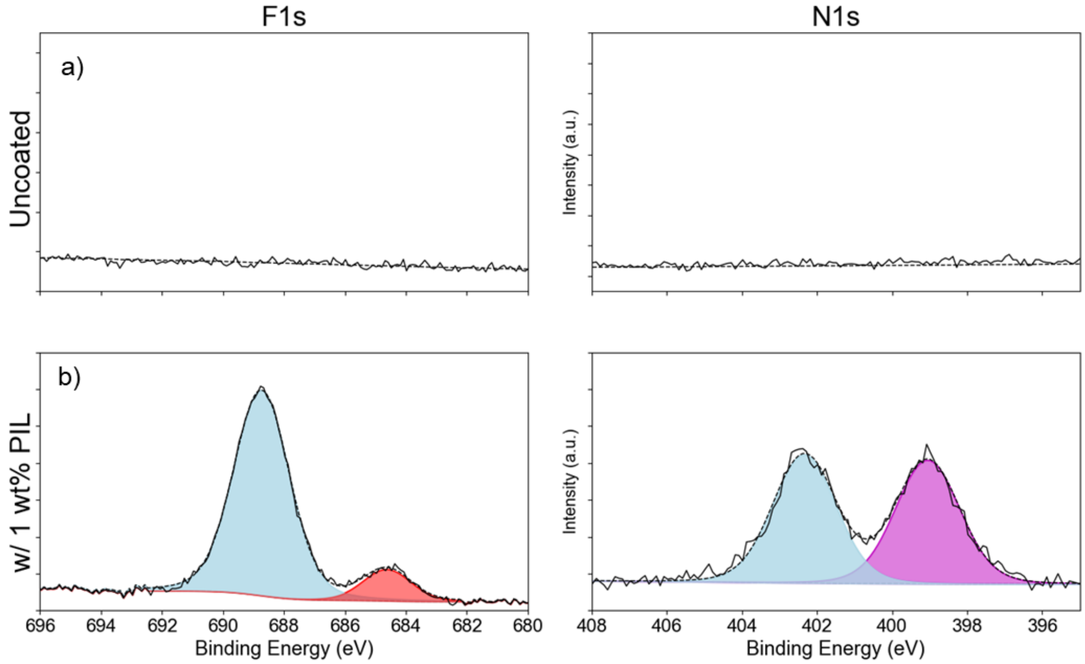

Figure S10: High resolution F1s and N1s XPS spectra of a) uncoated NMC82 powder and b) NMC82 powder coated with 1 wt% PIL

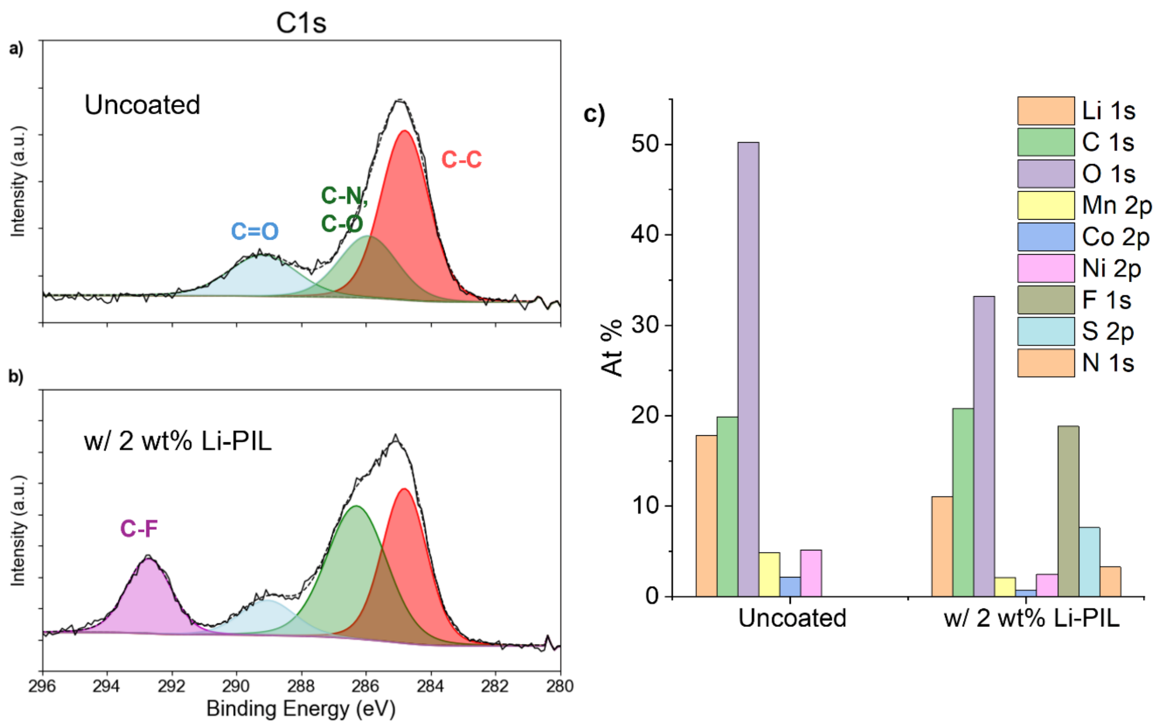

Figure S11: High-resolution C1s spectra of a) uncoated NMC82 and b) NMC82 with 2 wt% Li-PIL. c) XPS Survey elemental composition (in At%) of NMC82 and 2 wt% Li-PIL coated NMC82 powders

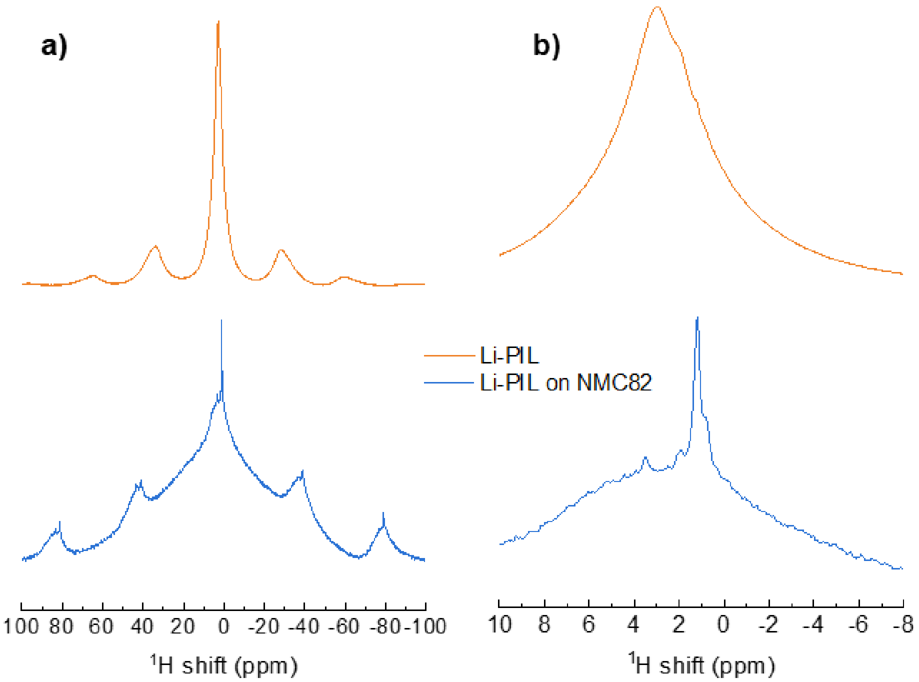

Figure S12: a) ^{1}H Hahn echo MAS NMR spectra of Li-PIL film (top) and NMC82 coated with Li-PIL (bottom). b) Zoomed inset of ^{1}H Hahn echo MAS NMR spectra of 2wt% Li-PIL film (top) and NMC82 coated with 2wt% Li-PIL (bottom)

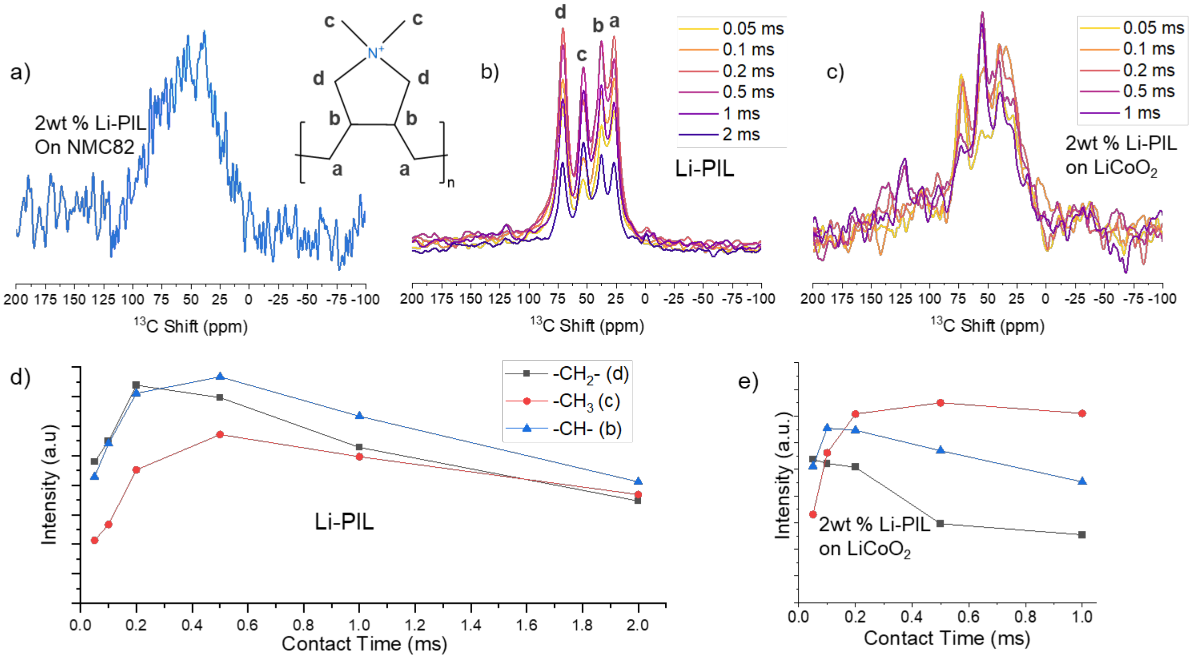

Figure S13: a) ^{1}H-^{13}C cross-polarization NMR spectra of 2wt% Li-PIL film (bottom) and NMC82 coated with 2wt% Li-PIL (top) at 15 kHz spinning speed b) ^{1}H-^{13}C CP NMR spectra of Li-PDDATFSI (Li-PIL) film at 10 kHz and contact times of 0.05 – 2 ms. c) ^{1}H-^{13}C CP NMR spectra of 2 wt% Li-PIL coated on $LiCoO_2$ at 10 kHz and contact times of 0.05 – 1 ms. d) Intensities of ^{13}C peaks as a function of contact time for the Li-PIL film e) Intensities of ^{13}C peaks as a function of contact time for 2 wt% Li-PIL coated on $LiCoO_2$.

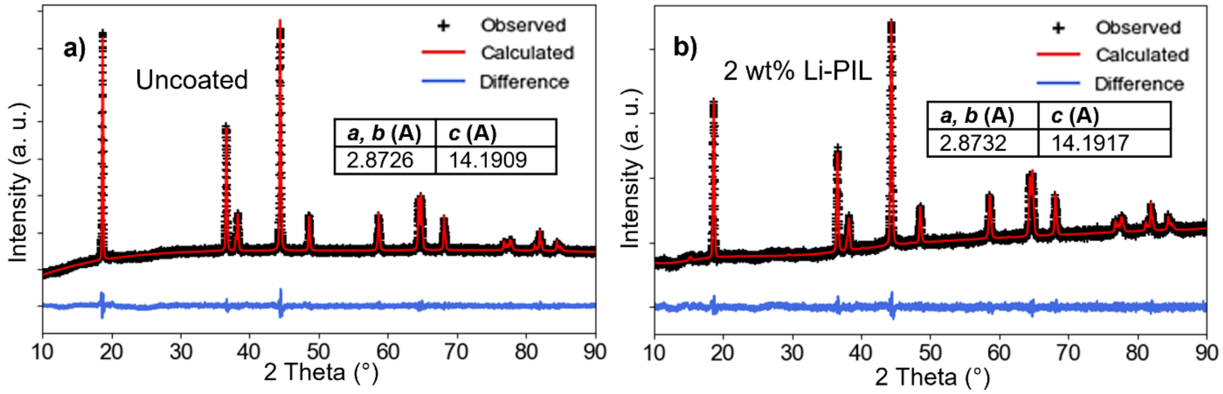

Figure S14: XRD spectra with Pawley refinement fit for a) uncoated NMC82 and b) NMC82 coated with Li-PIL

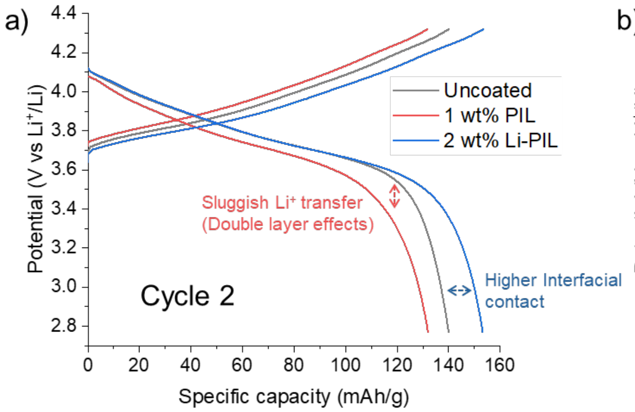

Figure S15: Comparison of charge-discharge voltage profiles for the uncoated, PIL and Li-PIL cells at 0.2C for a) 2nd and b) 100th cycles.

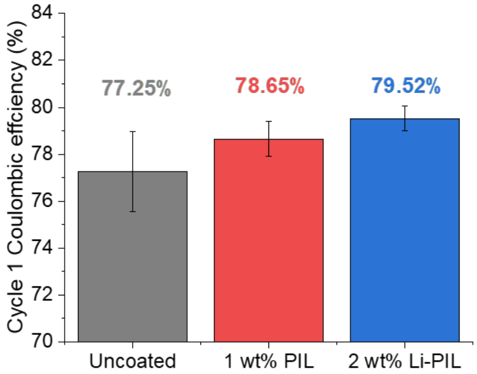

Figure S16: First cycle coulombic efficiencies for the uncoated, PIL and Li-PIL cells at 0.05C

Table S2: Cycle performance comparison of sulfide SSBs with Ni-rich and moderate-Ni cathodes reported by previous works and this work

Electrolyte	Coating and Active Material	CAM loading (mg/cm^2)	Temp. (°C)	Initial capacity @ 0.1C (mAh/g)	Cycles	Capacity retention	Ref.
Li_6PS_5Cl	SN:LiTFSI @ NMC83	10.69	25	168	100 @ 0.1C	>80%	[5]
Li_6PS_5Cl	PVBATFSI@ NMC83	10.69	25	~182	100 @ 0.1C	86%	[6]
Li_6PS_5Cl	DA@NMC85	8.97	25	190	200 @ 0.2C	90.4%	[7]
Li_6PS_5Cl	sPPSLi/PVP @ NMC90	10.6	25	186	152 @ 0.1C	71.6%	[8]
$Li_{9.54}Si_{1.74}P_{1.44}S_{11.7}Cl_{0.3}$	Al-GL-10 @NMC811	10.2	30	~190	100 @ 0.2C	88%	[9]
$Li_{10}GeP_2S_{12}$	cPAN-LATP @ NMC622	13.93	25	149.4	500 @ 0.5C	73%	[10]
$Li_{5.5}PS_{4.5}Cl_{1.5}$	LPSC@NMC85	~ 10.8	25	~180	100 @ 0.2C	84%	[11]
Li_6PS_5Cl	LTO@NMC70	10.39	25	135	100 @ 0.25C	48%	[12]
$Li_{10}GeP_2S_{12}$	$TiNb_2O_7$@NMC622	9.9	RT	180.3	140 @ 0.1C	92.2%	[13]
$Li_{10}GeP_2S_{12}$	$LiNbO_3$@NMC811	10.2	30	187	100 @0.3C	79.7%	[14]
$Li_{10}GeP_2S_{12}$	LPO@NMC811	8.9	RT	170.6	300 @0.2C	58.8	[15]
$Li_{10}GeP_2S_{12}$	LPOS@NMC811	8.9	25	161	250 @0.1C	80%	[16]
Li_6PS_5Cl	Li-PDDATFSI @NMC82	10	20	190	500 @ 0.2C	82.7%	**This Work**

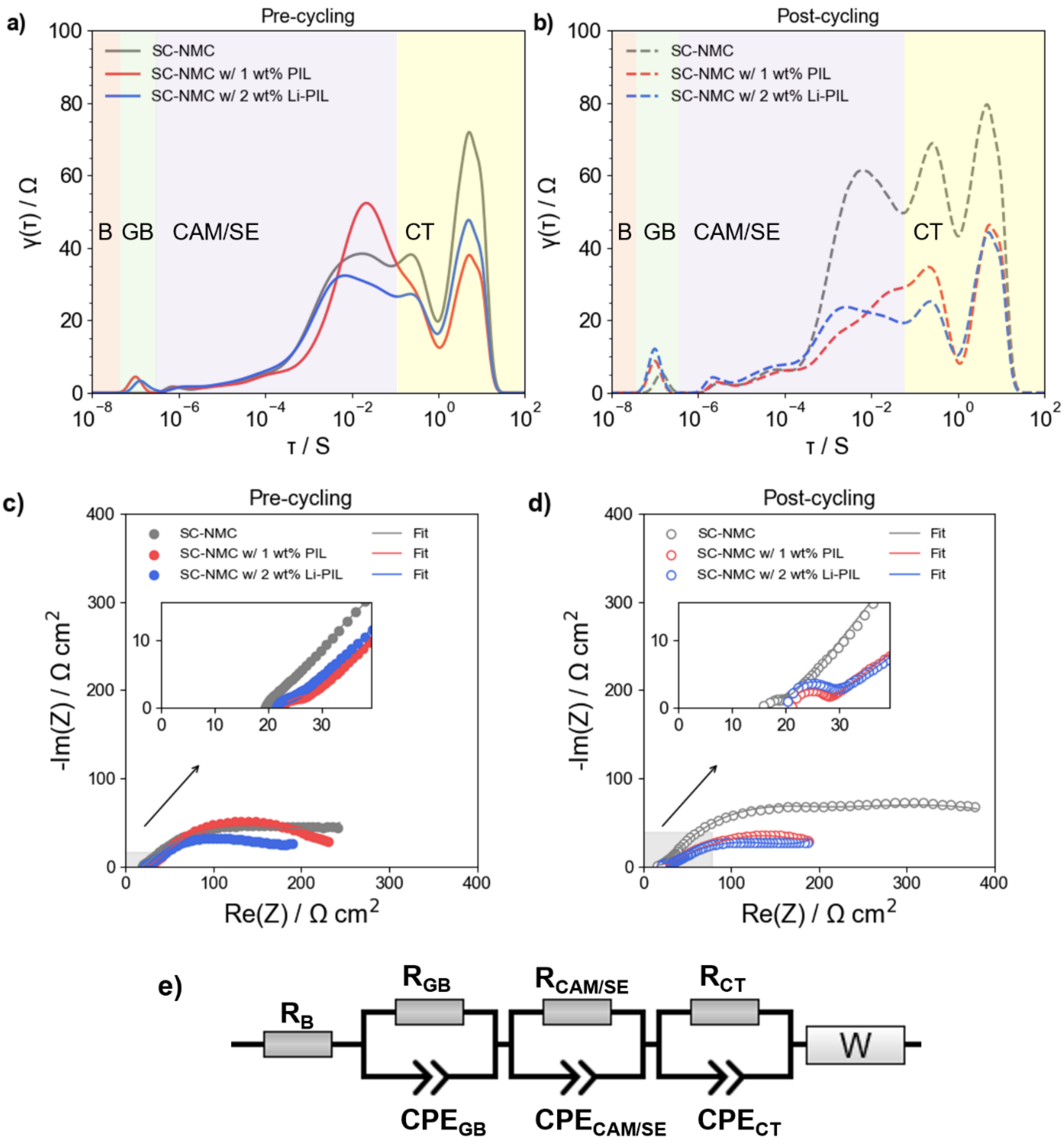

Figure S17: Top: Distribution of Relaxation Times (DRT) plots of a) pre-cycling and b) post-cycling for uncoated NMC82, NMC82 with a 1wt% PIL and 2wt% Li-PIL coatings. Middle: Top: Nyquist plots with fits for a) pre-cycling and b) post-cycling for uncoated NMC82, NMC82 with a 1wt% PIL and 2wt% Li-PIL coatings. Bottom: e) The equivalent circuit used for fitting the pre- and post-cycling Nyquist plots.

Table S3: Values of EIS equivalent circuit elements for uncoated and coated NMC82 cells, pre and post-cycling.

	**Uncoated Pre**	**Uncoated Post**	**1 wt% PIL Pre**	**1 wt% PIL Post**	**2 wt% Li-PIL Pre**	**2 wt% Li-PIL Post**
**R_B (Ω cm^2)**	19.27	15.25	22.44	20.17	21.18	19.21
**R_{GB} (Ω cm^2)**	1.45	3.92	3.82	7.37	3.13	8.44
**Q_{GB} (F/cm^2)**	8.60E-06	7.66E-06	6.97E-05	8.27E-07	5.19E-05	2.08E-07
**α_{GB}**	0.89	0.61	0.54	0.71	0.59	0.79
**$R_{CAM/SE}$ (Ω cm^2)**	91.42	113.10	52.76	57.12	59.04	63.73
**$Q_{CAM/SE}$ (F/cm^2)**	6.77E-04	1.97E-04	5.46E-03	1.73E-03	5.86E-04	1.17E-03
**$\alpha_{CAM/SE}$**	0.57	0.72	0.33	0.41	0.63	0.47
**R_{CT} (Ω cm^2)**	129.51	416.81	151.12	116.19	114.51	108.66
**Q_{CT} (F/cm^2)**	2.65E-03	2.05E-03	6.06E-04	2.32E-03	2.71E-03	3.18E-03
**α_{CT}**	0.44	0.39	0.64	0.52	0.38	0.33

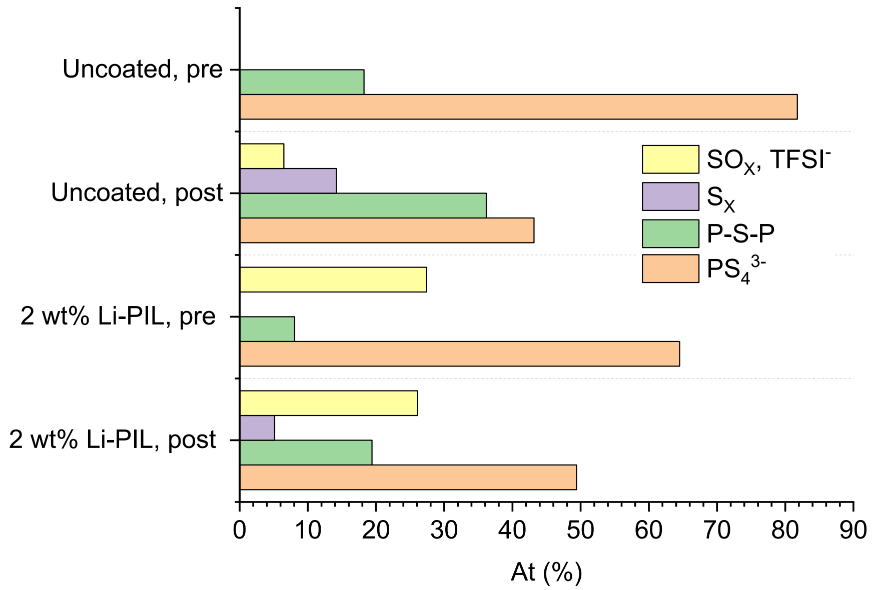

Figure S18: Comparison of pre and post-cycling atomic percentages of S-containing environments (normalized to total S 2p at%) for cathode composites with and without the Li-PIL coating

Table S4: Comparison of total corrected areas corresponding to TFSI-/SO_x environment in F 1s and S 2p spectra for the Li-PIL cathode composites

	Total Corrected Area, Raw Area/(RSF*T*MFP)	
**Name**	**2 wt% Li-PIL, pre**	**2 wt% Li-PIL, post**
**F 1s (TFSI$^-$)**	7732.49	8636.4
**S 2p (SOx, TFSI$^-$)**	5954.9	6319.68
**Ratio**	**1.30**	**1.37**

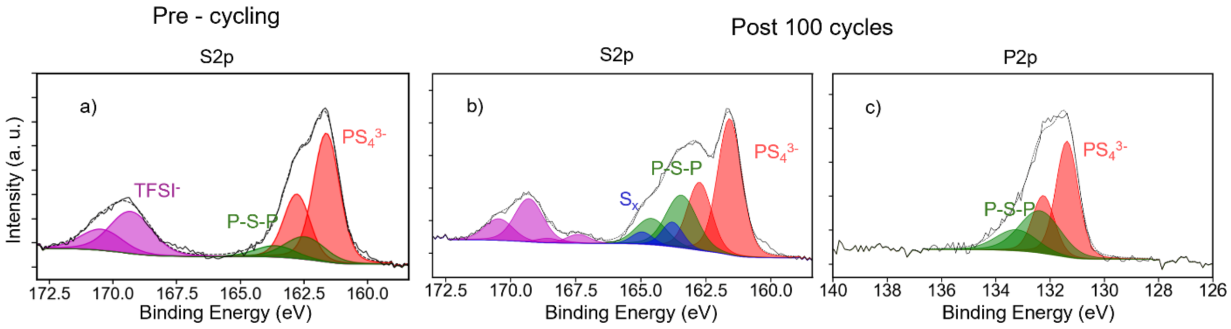

Figure S19: Pre-cycling S 2p and Post-cycling S 2p and P 2p XPS spectra of NMC82 cathode composite with 1 wt% PIL coating

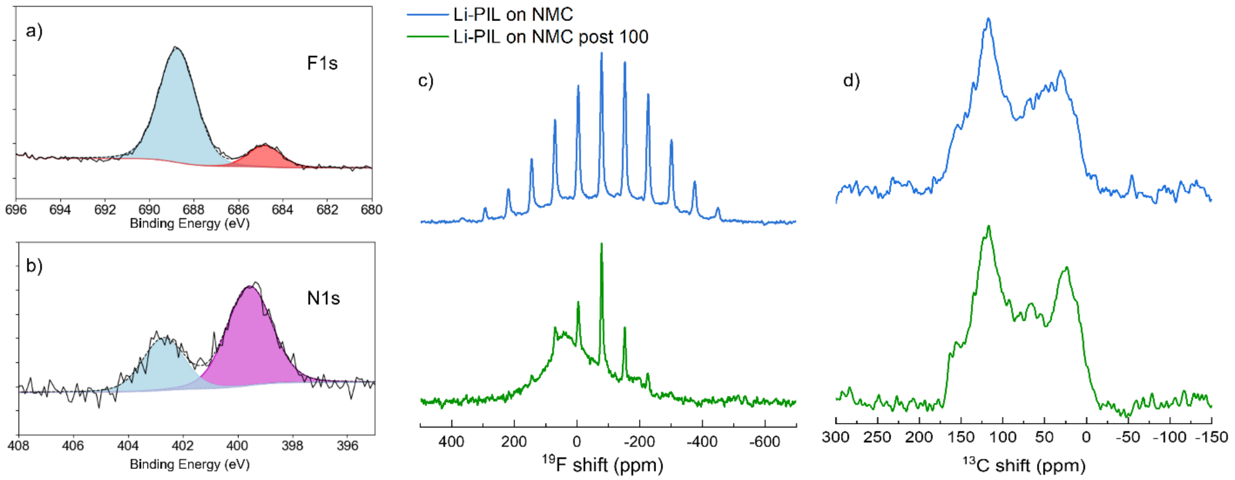

Figure S20: Post-cycling a) F 1s and b) N 1s XPS spectra of single-crystal NMC with 2wt% Li-PIL. c) ^{19}F MAS NMR spectra of the Li-PIL coated NMC82 cathode composite pre-cycling (top) and post 100 cycles (bottom) d) ^{13}C MAS proton decoupled NMR spectra of the Li-PIL coated NMC82 cathode composite pre-cycling (top) and post 100 cycles (bottom). The NMR spectra were acquired at a spinning speed of 35 kHz and a proton decoupling power of 30 W was used for the ^{13}C spectra

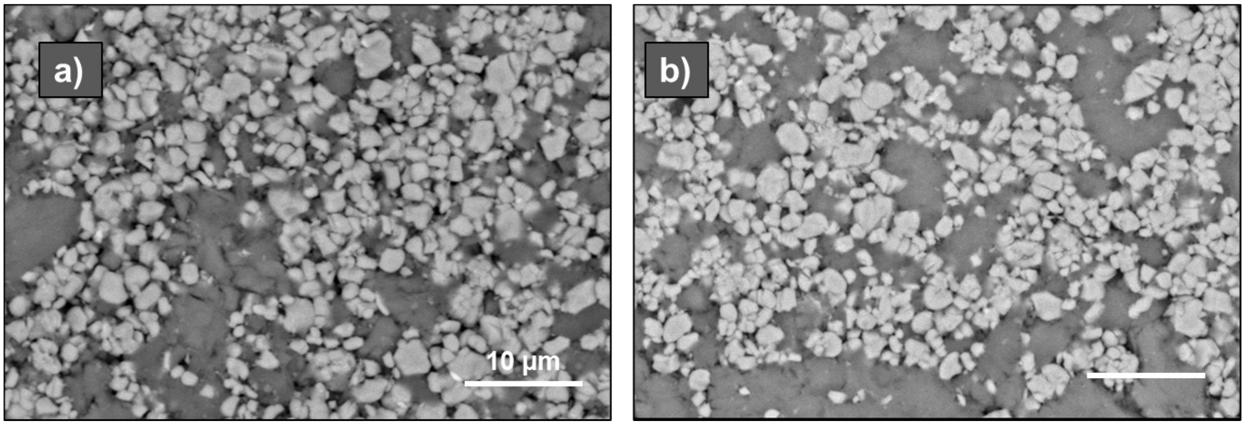

Figure S21: SEM images of the NMC82/LPSC composites with a) Uncoated NMC82 before cycling and b) NMC82 with 1 wt% PIL after cycling.

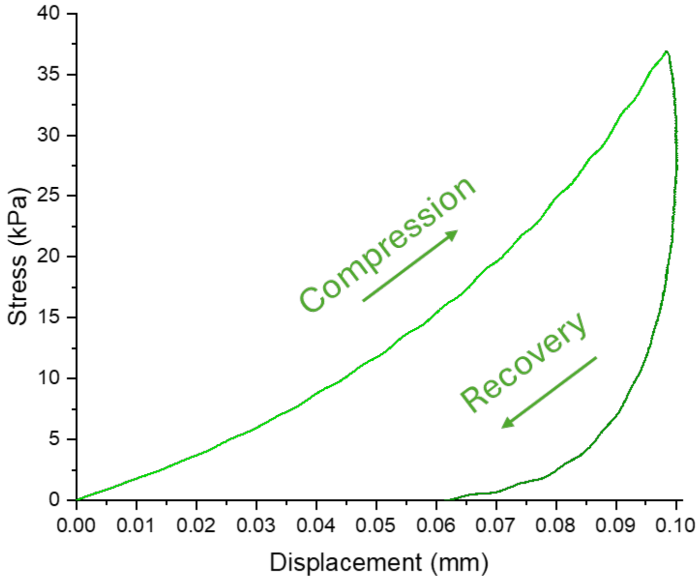

Figure S22: Stress-displacement curves of uniaxial compression test carried out on Li-PIL film at a rate of 2 µm/s.

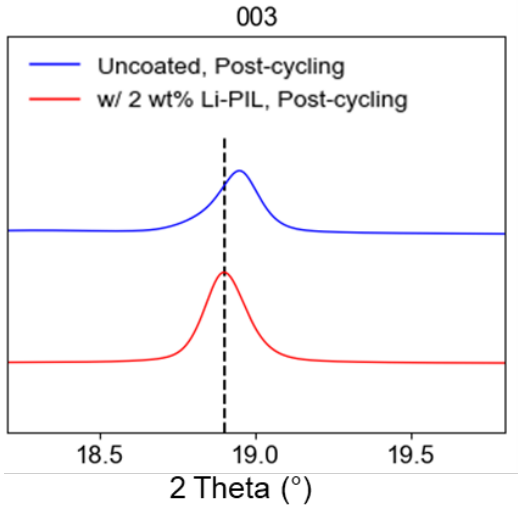

Figure S23: Zoomed inset of 003 peaks (of NMC82) for post-cycling uncoated NMC82, 2 wt% Li-PIL coated NMC82 cathode composites.

References:

(1) Vargas-Barbosa, N. M.; Roling, B. Dynamic Ion Correlations in Solid and Liquid Electrolytes: How Do They Affect Charge and Mass Transport? *ChemElectroChem* **2020**, *7* (2), 367–385. https://doi.org/10.1002/celc.201901627.
(2) Haverkort, W. *Electrolysers, Fuel Cells and Batteries: Analytical Modelling*; TU Delft OPEN Textbooks, 2024. https://doi.org/10.59490/tb.93.
(3) Wang, X.; Chen, F.; Girard, G. M. A.; Zhu, H.; MacFarlane, D. R.; Mecerreyes, D.; Armand, M.; Howlett, P. C.; Forsyth, M. Poly(Ionic Liquid)s-in-Salt Electrolytes with Co-Coordination-Assisted Lithium-Ion Transport for Safe Batteries. *Joule* **2019**, *3* (11), 2687–2702. https://doi.org/10.1016/j.joule.2019.07.008.
(4) Zhao, Q.; Bennington, P.; Nealey, P. F.; Patel, S. N.; Evans, C. M. Ion Specific, Thin Film Confinement Effects on Conductivity in Polymerized Ionic Liquids. *Macromolecules* **2021**, *54* (22), 10520–10528. https://doi.org/10.1021/acs.macromol.1c01820.
(5) Ma, R.; Liu, Y.; Fang, R.; Zhang, J.; Wang, Y.-H.; Huang, H.; Gan, Y.; He, X.; Xia, X.; Zhang, W.; Xia, Y.; Xin, S. A Plastic-Crystal Electrolyte Layer Promotes Interfacial Stability of Ni-Rich Oxide Cathode in Li6PS5Cl-Based All-Solid-State Rechargeable Li Batteries. *ChemSusChem* **2024**, *17* (24), e202400840. https://doi.org/10.1002/cssc.202400840.
(6) Shi, B.-X.; Yusim, Y.; Sen, S.; Demuth, T.; Ruess, R.; Volz, K.; Henss, A.; Richter, F. H. Mitigating Contact Loss in Li6PS5Cl-Based Solid-State Batteries Using a Thin Cationic Polymer Coating on NCM. *Adv. Energy Mater.* **2023**, *13* (24), 2300310. https://doi.org/10.1002/aenm.202300310.
(7) Huang, Y.; Zhou, L.; Li, C.; Yu, Z.; Nazar, L. F. Waxing Bare High-Voltage Cathode Surfaces to Enable Sulfide Solid-State Batteries. *ACS Energy Lett.* **2023**, *8* (11), 4949–4956. https://doi.org/10.1021/acsenergylett.3c01717.
(8) Shi, B.-X.; Weber, F.; Yusim, Y.; Demuth, T.; Vettori, K.; Münchinger, A.; Titvinidze, G.; Volz, K.; Henss, A.; Berger, R.; Richter, F. H. Lithiated Polymer Coating for Interface Stabilization in Li6PS5Cl-Based Solid-State Batteries with High-Nickel NCM. *J. Mater. Chem. A* **2025**, *13* (4), 2600–2614. https://doi.org/10.1039/D4TA07265K.
(9) Su, Y.; Liu, X.; Yan, H.; Zhao, J.; Cheng, Y.; Luo, Y.; Gu, J.; Zhong, H.; Fu, A.; Wang, K.; Wang, M.; Huang, J.; Yan, J.; Yang, Y. Assembly of an Elastic & Sticky Interfacial Layer for Sulfide-Based All-Solid-State Batteries. *Nano Energy* **2023**, *113*, 108572. https://doi.org/10.1016/j.nanoen.2023.108572.
(10) Liang, Y.; Liu, H.; Wang, G.; Wang, C.; Li, D.; Ni, Y.; Fan, L.-Z. Heuristic Design of Cathode Hybrid Coating for Power-Limited Sulfide-Based All-Solid-State Lithium Batteries. *Adv. Energy Mater.* **2022**, *12* (33), 2201555. https://doi.org/10.1002/aenm.202201555.
(11) Zuo, T.-T.; Walther, F.; Ahmed, S.; Rueß, R.; Hertle, J.; Mogwitz, B.; Volz, K.; Janek, J. Formation of an Artificial Cathode–Electrolyte Interphase to Suppress Interfacial Degradation of Ni-Rich Cathode Active Material with Sulfide Electrolytes for Solid-State Batteries. *ACS Energy Lett.* **2023**, *8* (3), 1322–1329. https://doi.org/10.1021/acsenergylett.2c02835.
(12) Negi, R. S.; Minnmann, P.; Pan, R.; Ahmed, S.; J. Herzog, M.; Volz, K.; Takata, R.; Schmidt, F.; Janek, J.; Elm, M. T. Stabilizing the Cathode/Electrolyte Interface Using a Dry-Processed Lithium Titanate Coating for All-Solid-State Batteries. *Chem. Mater.* **2021**, *33* (17), 6713–6723. https://doi.org/10.1021/acs.chemmater.1c01123.

(13) Sun, N.; Song, Y.; Liu, Q.; Zhao, W.; Zhang, F.; Ren, L.; Chen, M.; Zhou, Z.; Xu, Z.; Lou, S.; Kong, F.; Wang, J.; Tong, Y.; Wang, J. Surface-to-Bulk Synergistic Modification of Single Crystal Cathode Enables Stable Cycling of Sulfide-Based All-Solid-State Batteries at 4.4 V. *Adv. Energy Mater.* **2022**, *12* (29), 2200682. https://doi.org/10.1002/aenm.202200682.

(14) Liu, X.; Zheng, B.; Zhao, J.; Zhao, W.; Liang, Z.; Su, Y.; Xie, C.; Zhou, K.; Xiang, Y.; Zhu, J.; Wang, H.; Zhong, G.; Gong, Z.; Huang, J.; Yang, Y. Electrochemo-Mechanical Effects on Structural Integrity of Ni-Rich Cathodes with Different Microstructures in All Solid-State Batteries. *Adv. Energy Mater.* **2021**, *11* (8), 2003583. https://doi.org/10.1002/aenm.202003583.

(15) Deng, S.; Li, X.; Ren, Z.; Li, W.; Luo, J.; Liang, J.; Liang, J.; Banis, M. N.; Li, M.; Zhao, Y.; Li, X.; Wang, C.; Sun, Y.; Sun, Q.; Li, R.; Hu, Y.; Huang, H.; Zhang, L.; Lu, S.; Luo, J.; Sun, X. Dual-Functional Interfaces for Highly Stable Ni-Rich Layered Cathodes in Sulfide All-Solid-State Batteries. *Energy Storage Mater.* **2020**, *27*, 117–123. https://doi.org/10.1016/j.ensm.2020.01.009.

(16) Liang, J.; Zhu, Y.; Li, X.; Luo, J.; Deng, S.; Zhao, Y.; Sun, Y.; Wu, D.; Hu, Y.; Li, W.; Sham, T.-K.; Li, R.; Gu, M.; Sun, X. A Gradient Oxy-Thiophosphate-Coated Ni-Rich Layered Oxide Cathode for Stable All-Solid-State Li-Ion Batteries. *Nat. Commun.* **2023**, *14* (1), 146. https://doi.org/10.1038/s41467-022-35667-7.
